# Disrupting assembly of the inner membrane complex blocks *Plasmodium falciparum* sexual stage development

**DOI:** 10.1371/journal.ppat.1006659

**Published:** 2017-10-06

**Authors:** Molly Parkyn Schneider, Boyin Liu, Philipp Glock, Annika Suttie, Emma McHugh, Dean Andrew, Steven Batinovic, Nicholas Williamson, Eric Hanssen, Paul McMillan, Marion Hliscs, Leann Tilley, Matthew W. A. Dixon

**Affiliations:** 1 Department of Biochemistry and Molecular Biology, The University of Melbourne, Melbourne, Victoria, Australia; 2 Mass Spectrometry and Proteomics Facility, Bio21 Molecular Science and Biotechnology Institute, The University of Melbourne, Melbourne, Victoria, Australia; 3 Melbourne Advance Microscopy Facility, Bio21 Molecular Science and Biotechnology Institute, The University of Melbourne, Melbourne, Victoria, Australia; 4 Biological Optical Microscopy Platform, Bio21 Molecular Science and Biotechnology Institute, The University of Melbourne, Melbourne, Victoria, Australia; Bernhard-Nocht Institute for Tropical Medicine, GERMANY

## Abstract

Transmission of malaria parasites relies on the formation of a specialized blood form called the gametocyte. Gametocytes of the human pathogen, *Plasmodium falciparum*, adopt a crescent shape. Their dramatic morphogenesis is driven by the assembly of a network of microtubules and an underpinning inner membrane complex (IMC). Using super-resolution optical and electron microscopies we define the ultrastructure of the IMC at different stages of gametocyte development. We characterize two new proteins of the gametocyte IMC, called PhIL1 and PIP1. Genetic disruption of PhIL1 or PIP1 ablates elongation and prevents formation of transmission-ready mature gametocytes. The maturation defect is accompanied by failure to form an enveloping IMC and a marked swelling of the digestive vacuole, suggesting PhIL1 and PIP1 are required for correct membrane trafficking. Using immunoprecipitation and mass spectrometry we reveal that PhIL1 interacts with known and new components of the gametocyte IMC.

## Author summary

Transmission of the malaria parasite from humans to mosquitoes relies on the formation of the specialised blood stage gametocyte. *Plasmodium falciparum* gametocytes mature over about 10 days, during which time they undergo a remarkable morphological transformation, eventually adopting a characteristic crescent shape. The shape changes are thought to facilitate the mechanical sequestration of maturing gametocytes within the bone marrow and spleen, as well as the eventual release into the circulation. Failure to mature correctly leads to a failure to transmit. Despite the importance of this process, little is known about the molecular basis of elongation. In this work, we introduce 3D Electron Microscopy of *P*. *falciparum* gametocytes and use it, in a combination with super-resolution optical microscopy, to elucidate the genesis and expansion of the molecular structures that drive gametocyte elongation. We use protein interaction profiling to identify some of the proteins that help drive the shape change and employ inducible gene knockdown strategies to show that these proteins play a role in remodeling membranes, and are needed for gametocyte elongation. This work points to potential targets for the development of transmission-blocking therapies.

## Introduction

*Plasmodium falciparum*, the deadliest of the human malaria parasites, continues to impose an enormous economic and public health burden globally, resulting in about 480,000 deaths each year [1]. Nonetheless, considerable progress has been made in preventing and treating malaria infections and in targeting the mosquito vector, as evidenced by a decrease in mortality rates of more than 50% in some parts of Africa in recent years [1]. To cement these gains, there is an urgent need to target the transmission-competent sexual stage gametocyte, because asymptomatic carriers with persistent low level gametocytemia serve as a parasite reservoir during the low transmission season, leading to a resurgence of infections when mosquito numbers increase [2]. Transmission-ready *P*. *falciparum* gametocytes appear in the bloodstream 2–3 weeks after the first appearance of asexual stage parasites [3]. Ingestion of gametocytes by the *Anopheles* vector triggers their release from red blood cells (RBCs) and sexual reproduction in the mosquito gut. The resultant infectious sporozoites migrate to the salivary glands, and are transferred to the bloodstream of a new host when the mosquito next feeds.

*P*. *falciparum* gametocytes develop through five distinct stages over a period of 10–12 days as they prepare for transmission. Later stage gametocytes adopt a characteristic crescent or falciform shape unique to *P*. *falciparum* (see [4] for review). These shape changes are hypothesized to facilitate mechanical sequestration of developing gametocytes in privileged sites that include the bone marrow [5], thereby avoiding scrutiny and clearance from the circulation by the spleen. Upon maturation, stage V gametocytes release from their sites of sequestration and re-enter the circulation.

Gametocyte elongation is driven by a network of microtubules that assemble underneath flattened cisternal membrane compartments, known as the inner membrane complex (IMC) [6–8]. While related to the IMC of the invasive or motile stages (merozoite, ookinete and sporozoites), the gametocyte IMC has stage-specific functions that likely involve an as-yet poorly defined specialized set of proteins. Moreover, little is known about the genesis, development and organization of the supporting microtubule network and its interplay with the IMC.

To investigate the process of IMC assembly, we undertook a detailed ultrastructural survey of this organelle across the five stages of gametocyte development, highlighting the formation and expansion of the IMC plates as the gametocyte matures. We have investigated a previously identified IMC protein from *Toxoplasma gondii*, the Photosensitized 5-[125I] iodonaphthalene-1-azide Labeled protein-1 (PhIL1) confirming it as a component the IMC in *Plasmodium falciparum* [9]. Our experiments revealed the presence of PhIL1 at the IMC in very late stage schizonts and all stages of gametocyte development. We performed immunoprecipitation experiments with PhIL1-GFP parasites identifying eight interacting proteins, including three known IMC proteins and three new putative IMC proteins. The top **P**hIL1 **I**nteracting **P**rotein, PIP1, localizes to the IMC, but shows a more restricted localization than PhIL1 in stage IV gametocytes. Functional characterization of PhIL1 and PIP1 by gene knockdown show that upon depletion of either of these proteins, gametocyte elongation is ablated and the gametocytes fail to reach maturity. The IMC of knockdown parasites is under-developed and they exhibit a swollen digestive vacuole, indicating a role for PhIL1 and PIP1 in the membrane trafficking events that drive IMC genesis, expansion and gametocyte elongation.

## Results

### Plasmodium gametocytes lack apical-basal polarity

**Fig 1:**
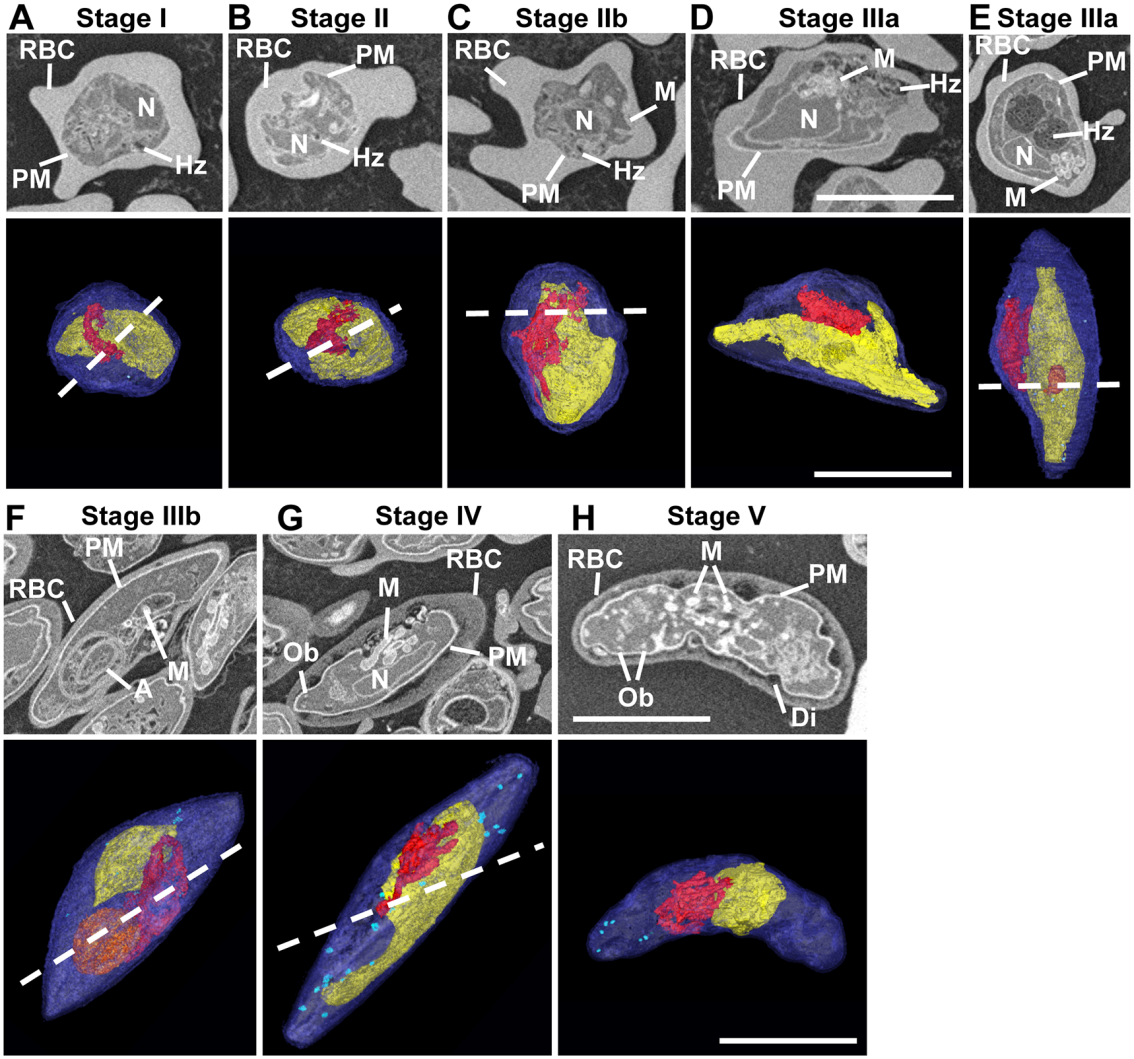
Whole cell reconstructions reveal organelle organization during gametocyte development. (A-H) Individual SBF-SEM images of different stages (top rows), and rendered images of serial SBF-SEM sections (bottom rows). The following structures are labeled in the sections and color-coded in the rendered images. Parasite plasma membrane/parasitophorous vacuole membrane (PM, blue), mitochondrion (M, red), nucleus (N, yellow), hemozoin (Hz), the apicoplast (A, orange), osmophillic bodies (OB, light blue), Dimples (Di) and RBC. Scale bars: 5 μm. The dotted line marks the plane of sectioning. Rotations of these models and translations through the SBF-SEM images can be seen in S1–S6 Videos. Quantitative measurements and relative organelle positions are presented in S1 and S2 Figs and S1 Table.

Serial block-face scanning electron microscopy (SBF-SEM) generates high-resolution 3D images of larger sample volumes at ~50 nm resolution [10, 11]. We employed SBF-SEM to obtain a detailed map of membrane organization and organelle placement across gametocyte development (Fig 1). The cellular features are revealed in individual SBF-SEM “sections” (Fig 1 top panels). Rendering of these features in successive sections provides a 3D view of parasite ultrastructure (Fig 1 bottom panels). Features such as the RBC membrane (RBC), parasite plasma membrane/parasitophorous vacuole membrane (PM, blue) and nucleus (N, yellow) are readily identified. From late stage II gametocytes the mitochondrion (M, red) can be seen in addition to the apicoplast (A, orange) (Fig 1D–1H). Mature stage IV and V gametocytes have osmophillic bodies (Ob, light blue) (Fig 1G and 1H). These are best appreciated by viewing the translations through the SBF-SEM sections (S1–S3 Videos) and the 3D rotations of the 3D rendered images (S4–S6 Videos). Parasite elongation (length to width ratio) reached a maximum value (4.6) at stage IV, and then decreased (3.3) at stage V. Nuclear elongation reached a maximum value (3.3) at stage III, then decreased at stage V (1.2). Mitochondrial elongation reaching a maximum value (3.5) at stage IV, then decreased at stage V (1.4) (S1 Fig). Analysis of 35 individual stage III/IV gametocytes revealed no evidence for apical-basal polarity, as is observed in the invasive or motile stages of development (merozoites, sporozoites and ookinetes) where the IMC is also present. That is, there is no apical complex, the nucleus was roughly centrally located, and we observed a roughly even distribution of the mitochondrion and digestive vacuole complex with respect to the two ends and two sides of the cell (Supp S2 Fig, S1 Table).

### The IMC expands via addition of membrane to the leading edge of the plates

**Fig 2:**
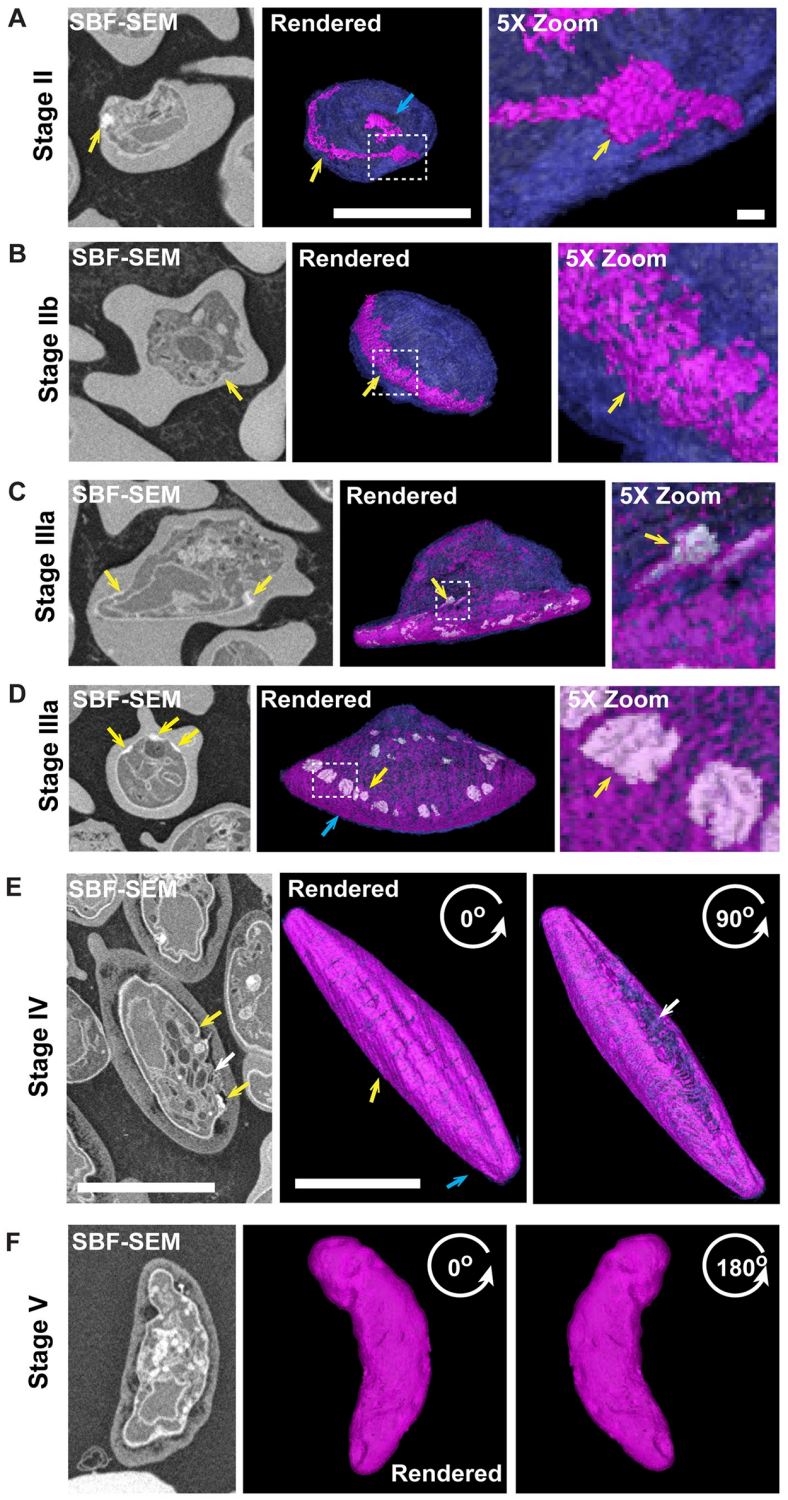
The IMC is expanded from the leading edge. (A-F) SBF-SEM showing the organization and genesis of the IMC plates at the gametocyte periphery. Rendered models (right) and individual SBF-SEM sections (left) are shown. The IMC is identified as an electron-scattering structure at the parasite periphery. The rendered 3D models reveal (A) a narrow semi-circular strip of thickened membrane at the periphery of stage II gametocytes (yellow arrow), transitioning (B) to a ribbon of disc-like plates in stage IIb (yellow arrow). (C-D) In stage III of development, these plates have expanded. Regions of thickening (yellow arrow, rendered in white) are observed at the leading edges of these plates. These may represent regions where new membrane is being deposited. (E) The plates of the IMC are clearly visible in stage IV gametocytes (yellow arrow). (F) Stage V gametocyte showing the relaxed IMC membrane. Rendered models show the Parasite Plasma Membrane (PPM, blue) and the Inner Membrane Complex (IMC, magenta). Scale bars: 5 μm. 5x Zoom images are shown on the right. Scale bars: 1 μm. SBF-SEM sections and rotations of the rendered models are presented in S1–S6 Videos.

We next investigated IMC formation by SBF-SEM. The phospholipid-rich (double membrane) nature of the IMC promotes binding of the reduced osmium stain, thus permitting its detection by intensity thresholding and semi-automatic segmentation (Fig 2; see S1–S6 Videos). In early stage II gametocytes, regions of thickened membranes are observed (Fig 2A, SBF-SEM section, yellow arrow). Rendering of this structure reveals a narrow semi-circular strip of thickened membrane at the parasite periphery (Fig 2A, purple, yellow arrow). Pockets of deposited membrane material are also observed in other regions of the cell (Fig 2A, blue arrow). The zoom image shows the formation of a disk-like structure (Fig 2A, zoom, yellow arrow). As the parasite elongates to the “lemon-shaped” stage IIb, the initial line of membrane expands to form a ribbon (Fig 2B, yellow arrow). The striped appearance of the IMC is already evident at this stage (Fig 2B zoom, yellow arrow IMC plates). By stage III, the IMC begins to expand and wraps further around the parasite (Fig 2C and 2D). Regions of extra thick membrane are evident along the growing edge of the IMC (Fig 2C and 2D, yellow arrow and zooms). These membrane thickenings are seen within the SBF-SEM sections (Fig 2C and 2D, yellow arrows). This suggests that the IMC plates are extended laterally by deposition of membrane material at the leading edge. By stage IV, the parasite is almost completely enclosed by the IMC and elongation is most pronounced (Fig 2E). Rotations at 0 and 90° of the rendered model highlight the structure of the IMC plates and the expansion of the IMC structure (Fig 2E, yellow arrows). These rotations illustrate the opening where the IMC plates have not fused (Fig 2E, 90° white arrow and see S6 Video for rotations). Our 3D-SIM analysis revealed that the IMC is made up of 13 cisternal plates in total of which the two end plates are larger. Examination of the SBF-SEM reveal that the 11 internal plates (Fig 2E, yellow arrow) have an average width of 0.85 ± 0.27 μm, while the 2 plates near the tips of the gametocytes (Fig 2E, blue arrow) have an average width of 2.2 ± 1.6 μm. By stage V the ends of the parasite are rounded and the IMC stripes are no longer evident in SBF-SEM (Fig 2F), potentially due to a relaxing of the gametocyte upon disassembly of the microtubule network. Indentations of the parasite surface and IMC, which occur during fixation, may represent areas of weakness in the IMC and parasite plasma membrane, as the microtubule skeleton is disassembled, in the mature stage V parasite (S3 and S6 Videos).

### PhIL1 is a component of the IMC in *P*. *falciparum*

**Fig 3:**
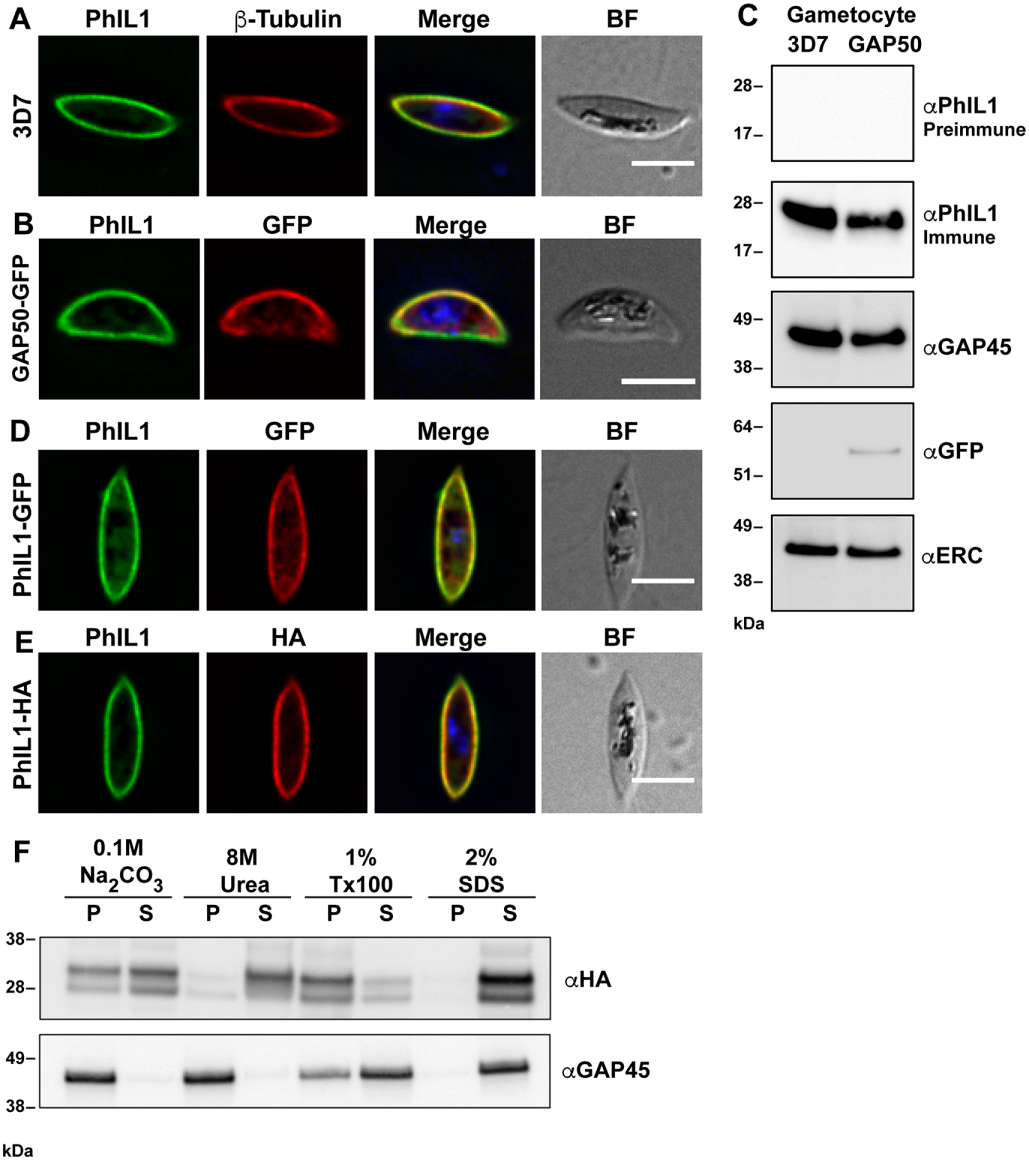
PhIL1 is located at the IMC in gametocytes. (A) Immunofluorescence microscopy showing anti-PhIL1 (green) at the periphery of a 3D7 stage IV gametocyte, showing fluorescence close to the anti-β-tubulin (red) labeling. (B) Immunofluorescence microscopy of a stage IV gametocyte showing overlap of GAP50-GFP (red) and PhIL1 (green) at the periphery of the cell. (C) Western blot analysis of saponin-treated pellets of 3D7 and GAP50-GFP stage IV gametocytes. Gametocytes were probed with PhIL1 pre-immune serum, anti-PhIL1 antiserum, anti-GAP45, anti-GFP and anti-ERC. (D, E) Immunofluorescence microscopy of stage IV PhIL1-GFP (D) and PhIL1-HA (E) gametocyte transfectants. Parasites were labeled with anti-PhIL1 (green) and anti-GFP or anti-HA (red). Nuclei were labeled with DAPI. Scale bars: 5 μm. (F) Purified, saponin-lysed PhIL1-HA gametocytes were solubilized with different extraction agents for 30 min. Pellet and supernatant fractions were separated and loaded in equivalent amounts on 4–12% acrylamide gels. PhIL1 and GAP45 bands were visualized using mouse anti-HA or rabbit anti-GAP45 primary and HRP-conjugated goat anti-mouse or goat anti-rabbit secondary antibodies, respectively. P = pellet; S = supernatant. Western analysis and immunofluorescence of PhIL1 is presented in S3 Fig.

To further study IMC genesis and organization, we characterized the *P*. *falciparum* homologue (PF3D7_0109000.1) of *T*. *gondii* PhILl, which shares 56% sequence identity within the C-terminal region (amino acids 130–217). S3 Fig provides a protein alignment of the sequences for a number of PhIL1 homologues in EupathDB. The N-terminal region is highly divergent and contains a motif (amino acids 17–91) that is found in non-muscle myosins. To study the cellular location of PhIL1 a recombinant protein corresponding to full-length PhIL1 (predicted molecular mass of 25,477 Da), was expressed in *E*. *coli* and used to generate rabbit polyclonal antiserum. Immunofluorescence microscopy of stage IV gametocytes revealed a strong PhIL1 signal at the periphery, where it is closely associated with β-tubulin (Fig 3A) and was located close to the known IMC marker, the glideosome-associated protein 50 (GAP50) (Fig 3B). Likewise, PhIL1 was found at the IMC in late stage schizonts (S4A and S4B Fig). The specificity of the immune serum is indicated by its recognition of a single band of ~28 kDa in Western blots of the pellet fraction of saponin-permeabilized gametocytes (3D7 WT and GAP50-GFP transfectants, Fig 3C). No bands were observed when protein extracts were probed with pre-immune sera (Fig 3C). This sample was also positive for the IMC marker, GAP45 (Fig 3C). Probing with anti-GFP confirmed the correct expression of the GAP50-GFP chimeric protein and the ER marker *Pf*ERC was used as a loading control (Fig 3C; see S10 Fig for full-length blots).

To further investigate the location of PhIL1 in *P*. *falciparum* we generated transgenic parasites in which full length GFP-tagged PhIL1 was episomally expressed under the control of the gametocyte-specific Pfs16 promoter (PhIL1-GFP) [12]. A PhIL1-HA line was also generated by integrating 3xHA into the 3’ end of the *PhIL1* locus. Immunofluorescence signals for HA and GFP overlapped with the signal from anti-PhIL1 antibody, confirming the correct location of the tagged proteins (Fig 3D and 3E). Western blotting of extracts of transfected gametocytes with anti-PhIL1, anti-GFP and anti-HA revealed the expected bands of 28–30 kDa in the PhIL1-HA line (which sometime appeared as a doublet), and 48 kDa in the PhIL1-GFP line (S4C Fig). Both samples were positive for *Pf*ERC (S4C Fig).

The PhIL1 antiserum recognizes a band of ~28 kDa in schizont stage asexual parasites, plus a possible breakdown product at ~20 kDa (S4C Fig). Immunofluorescence microscopy revealed a signal at the periphery of merozoites in very late stage schizont-infected RBCs and in free merozoites (S4A and S4B Fig), colocating with the IMC marker GAP45 (S4B Fig). No PhIL1 signal was observed in ring and trophozoite-infected RBCs and no fluorescence signal was observed with the pre-immune serum.

Saponin-permeabilized PhIL1-HA gametocytes were subjected to solubility profiling using different extraction agents. PhIL1 was only partially extracted with carbonate buffer and Triton X-100, but fully extracted in 8M urea and 2% SDS. This is suggestive of an association with the cytoskeleton and peripheral association with the IMC (Fig 3F). Blots were re-probed with anti-GAP45 to validate the solubility profile (Fig 3F) [9].

### Arrangement and organisation of the IMC membrane plates and microtubules at different stages of gametocyte development

**Fig 4:**
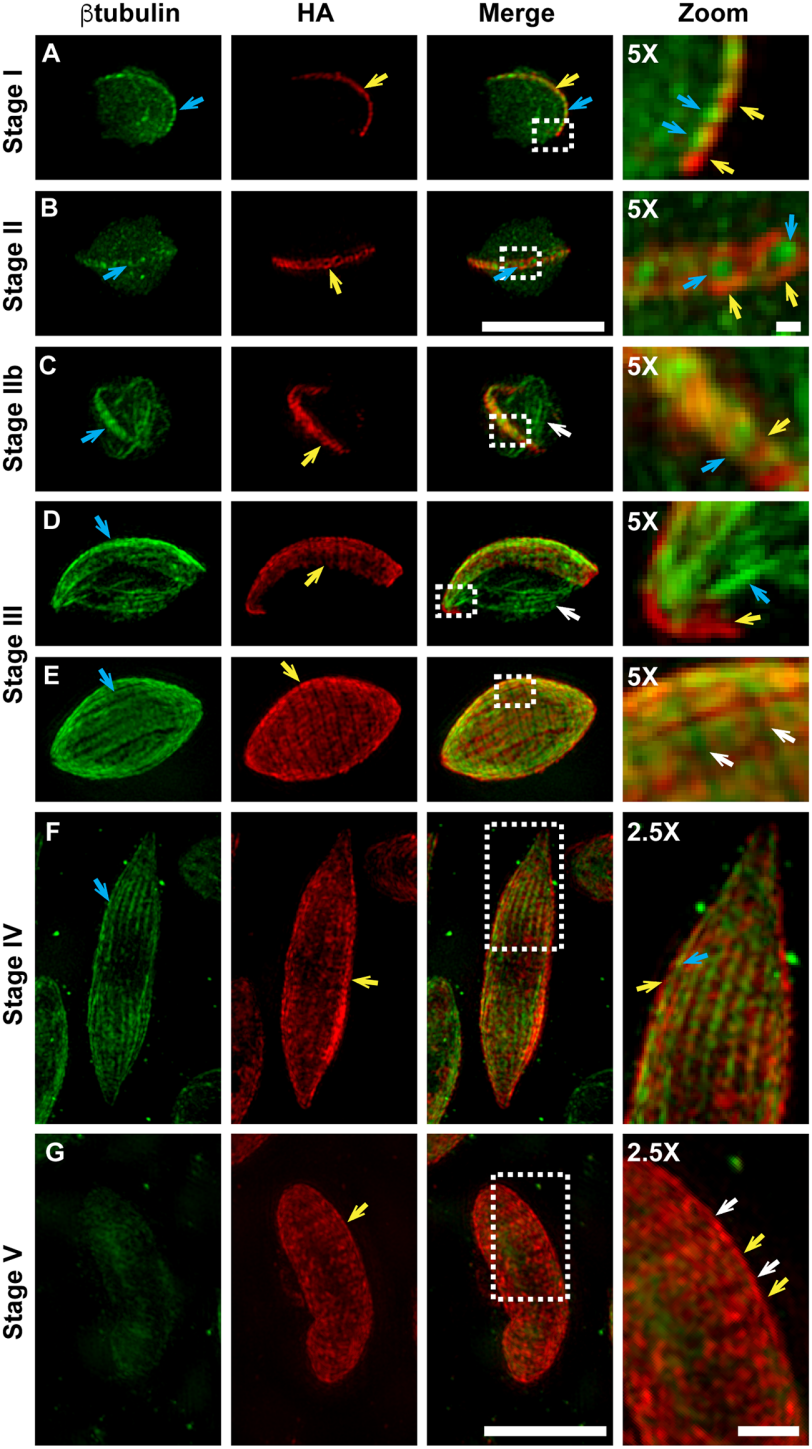
Genesis and development of the gametocyte IMC. (A-G) 3D-SIM immunofluorescence microscopy reveals the location of PhIL1-HA (red) relative to β-tubulin (green) for PhIL1-HA gametocytes from stage I-V of development. (A) A row of PhIL1-HA labeled puncta (yellow arrow) is observed along one edge of the parasite, interleaved with β-tubulin staining (blue arrow). (B) In stage II gametocytes, PhIL1-HA is observed as 13 ring-like structures (yellow arrows) aligned at the periphery of the parasites. Accumulations of β-tubulin staining can be seen in the centers of these disks (blue arrow). (C) The nascent IMC plates are more homogenously labeled with PhIL1-HA (yellow arrow), forming a ribbon-like structure at the periphery of the cell. Microtubules align with the IMC plates (blue arrow) or cross the parasite cytoplasm (white arrow). (D-E) In stage III, the IMC plates are clearly defined with homogenous PhIL1-HA labeling (yellow arrow). Bundles of microtubules underlie the IMC (blue arrow) or cross the parasite cytoplasm (white arrow). (F) In stage IV gametocytes, the IMC largely encases the parasite. The PhIL1-HA plates (yellow arrow) are still clearly visible and the microtubule network is tightly associated with the IMC (blue arrow). (G) By stage V, the microtubule network is disassembled but the IMC (labeled with PhIL1-HA) remains at the parasite periphery (yellow arrows; sutures are indicated with white arrows). 5x and 2.5X zoom images are shown on the right hand side. Scale bars: 5 μm. Scale bars: zoom images: 1 μm. The represented area is marked on the images with a white box. Rotations of these 3D-SIM images are provided in S7 and S8 Videos.

To investigate the formation and expansion of the IMC we made use of the PhIL1-HA parasite line and performed 3D-SIM super-resolution microscopy at different stages of early gametocyte development. The initial phase of IMC formation (in stage I) is associated with the deposition of a semi-circular ribbon of PhIL1 at the parasite periphery (Fig 4A, yellow arrow). Co-staining with anti-β-tubulin antiserum reveals punctate buds that form an alternating pattern within the PhIL1 ribbon (Fig 4A, blue arrow). Puncta of β-tubulin are also observed in other regions of the cell; these may represent microtubule “seeds” (Fig 4A). The spatial organization of the PhIL1 and β-tubulin puncta are best appreciated in the zoom in images (Fig 4A, zoom) and in the 3D rotations of the 3D-SIM images (S7 Video). As the cell transitions to stage II, the PhIL1 puncta form 13 small circular structures that represent the nascent IMC plates (Fig 4B, yellow arrow. S7 Video). An accumulation of β-tubulin is observed at the center of each of these nascent plates (Fig 4B, blue arrow, zoom). The data indicate that the nascent IMC plates may stabilize microtubule nuclei, thereby promoting microtubule polymerization and growth; however it is also possible that IMC assembly is driven by microtubule polymerization.

As the gametocyte develops further, bundles of microtubules extend along the nascent plates (Fig 4C, blue arrow). In addition, extended microtubules are observed traversing the parasite cytoplasm (Fig 4C, white arrow). Electron microscopy analysis of freeze-substituted stage II-III gametocytes confirms that microtubules lie closely opposed to the IMC (S5A Fig, yellow arrows) as well as crossing the cytoplasm (S5B Fig, blue arrows).

In stage III gametocytes, the IMC plates expand laterally (Fig 4D and 4E, yellow arrow). The leading edge of the IMC can be seen at the tip of the gametocyte with closely associated β-tubulin (Fig 4D, zoom). The parasite starts to elongate due to the formation of extended microtubules underneath the IMC plates (Fig 4D and 4E, blue arrow) and in the regions away from the IMC plates crossing the parasite cytoplasm (white arrow). The organization of the IMC plates, the sutures (white arrows) and microtubules can be seen in the zoom image of Fig 4E.

The expansion of the IMC plates is completed in stage IV where they almost cover the entire parasite, with a small opening present where the plates have not yet fused (Fig 4F; best appreciated by examining the rotation of the 3D-SIM images; S8 Video). The stage IV microtubules are all tightly associated with the IMC, forming a dense network (Fig 4F, blue arrow, zoom). Electron microscopy reveals long continuous microtubules running the length of the gametocyte (S5E Fig, blue arrows) and arranged in a tightly packed array at the tips of the stage IV gametocyte (S5E and S5F Fig, yellow arrows).

In stage V of development, the microtubule skeleton is depolymerized, leaving only a weak, diffuse labeling with anti-β-tubulin (Fig 4G). The plates of the IMC are still visible delineated by the darker suture lines (Fig 4G, zoom). Electron microscopy of stage V gametocytes reveals the presence of small microtubule stubs bound to the IMC, often in small bundles (S5G and S5H Fig, yellow arrows). This depolymerisation of the microtubules coincides with the previously reported increase in cellular deformability at stage V of development [13, 14].

### Genetic attenuation of PhIL1 arrests gametocyte development

**Fig 5:**
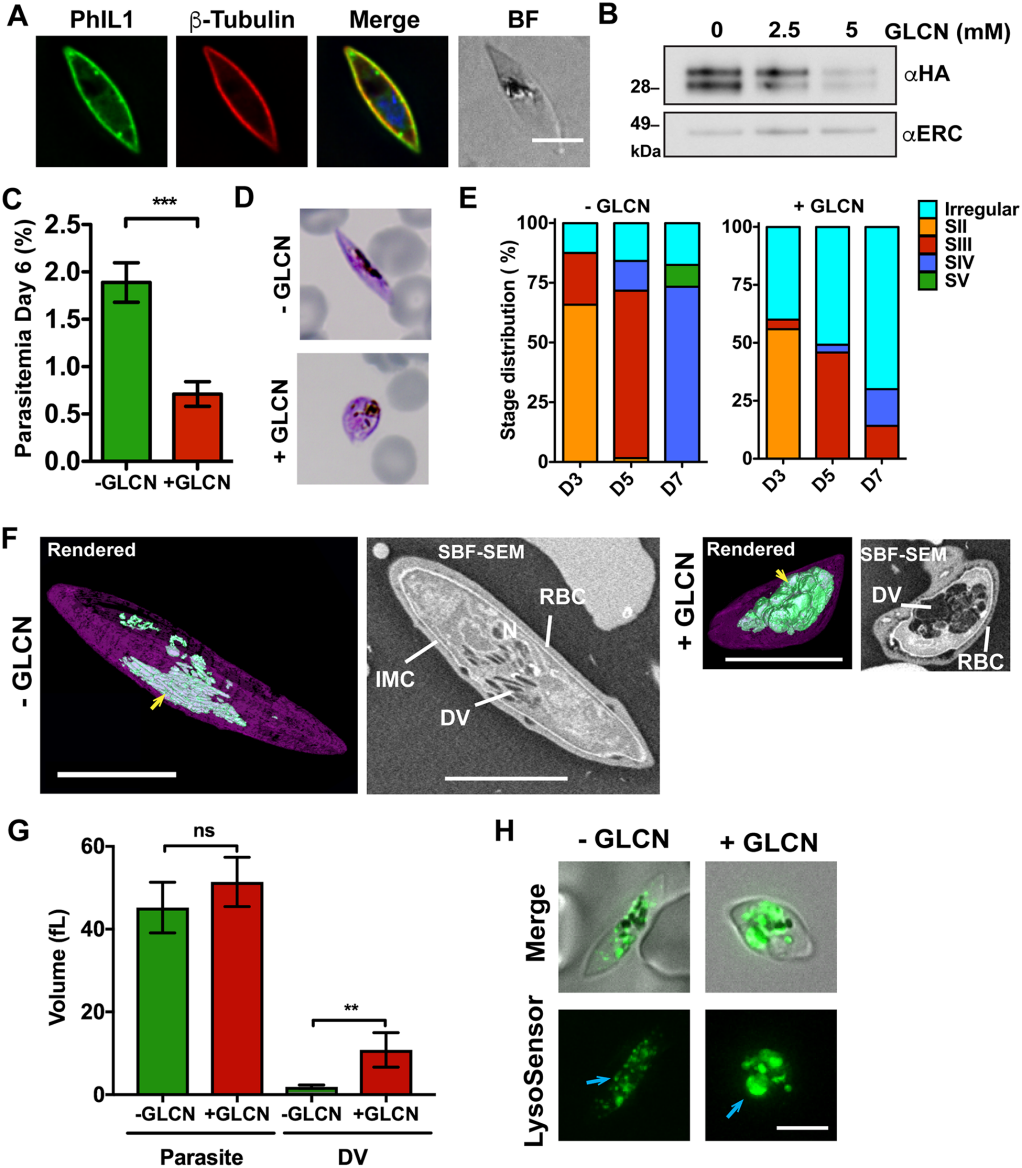
Knockdown of PhIL1 arrests gametocyte development. (A) PhIL1-HA-*glmS* (green) is located at the parasite periphery, closely associated with β-tubulin (red). (B) Stage IV PhIL1-HA-*glmS* gametocytes treated with a range of glucosamine concentrations and analyzed by Western blotting. Probing with anti-HA shows efficient knockdown of the PhIL1-HA protein when compared to the anti-*Pf*ERC loading control. (C) Glucosamine-treated (+GLCN, 5 mM) and untreated (-GLCN, 0 mM) cultures were induced to form gametocytes and the parasitemia estimated at day 6. Data represent mean ± SEM; n = 3 experiments performed in triplicate. *** P <0.001, unpaired t-test. (D) Representative Giemsa-stained smears highlighting the altered morphology following PhIL1 knockdown. (E) Stage progression counts for PhIL1-HA-*glmS* parasites plus or minus glucosamine, highlighting the arrest in development following knockdown. Days 3, 5 and 7 only are shown. The complete data set, including wild type parasites plus or minus glucosamine, are presented in S6C and S6D Fig. Data from 2 separate experiments performed in triplicate. Mean values are shown. (F) SBF-SEM images of PhIL1-HA-*glmS* parasites, plus or minus glucosamine. Rendered models are shown on the left, highlighting the IMC in purple and the digestive vacuole in green. A swollen digestive vacuole (DV; yellow arrow) is observed in glucosamine-treated parasites. Scale bars: 5 μm. (G) Quantification of the SBF-SEM images. Mean volumes for the parasite and the digestive vacuole are shown. Data represent mean ± SEM. n = 5. ** P <0.01, unpaired t-test. (H) PhIL1-HA-*glmS* parasites plus or minus glucosamine were labeled with LysoSensor to highlight the acidic digestive compartments. Swollen compartments are seen in the treated group, but not in the untreated control group (blue arrows).

To assess the function of PhIL1, we generated an inducible gene knockdown using the *glmS* riboswitch system [15, 16]. Integration of the *PhIL1-HA-glmS* plasmid into the endogenous PhIL1 locus in NF54 parasites was confirmed by PCR (S6A and S6B Fig). Immunofluorescence microscopy confirmed the location of PhIL1-HA at the gametocyte periphery where it overlaps with β-tubulin (Fig 5A). Treatment of PhIL1-HA-*glmS* parasites with glucosamine was performed for 6 days from a ring stage culture containing both asexual and gametocyte rings. Apparent knockdown of PhIL1-HA to a level of 85% (by densitometry) was achieved with 5 mM glucosamine (Fig 5B). Knock-down was associated with a 62% reduction (t-test, P = 0.001) in the numbers of gametocytes (Fig 5C) and the remaining gametocytes had significantly altered morphology (Fig 5D and 5E, S6D Fig). In wildtype NF54 parasites or in untreated PhIL1-HA-*glmS* parasites, more than 75% of the parasites reach stage IV/V by day 7 (Fig 5E, S6C Fig), while less than 15% of the remaining gametocytes in the treated PhIL1-HA-*glmS* line have progressed morphologically past stage III (Fig 5E). Untreated PhIL1-HA-*glmS* transfectants reached an average cell length of 10.3 ± 0.3 μm by day 6, similar to the treated or untreated NF54 (S6F Fig). By contrast, glucosamine-treated PhIL1-HA-*glmS* parasites had an average length of only 4.9 ± 0.4 μm. Taken together these data indicate that PhIL1 is needed for gametocytes to progress morphologicaly past stage III of development. By contrast, treatment of asexual stage parasites with 5 mM glucosamine had no effect on parasite growth (S7A Fig), demonstrating that PhIL1 is not needed (or needed only at low level) for asexual reproduction. Immunofluorescence analysis of the late stage marker Pfs48/45 indicated that this protein in produced in the few PhIL1 knock-down parasites that survive treatment with glucosamine (S7B Fig).

Immunofluorescence microscopy of PhIL1-HA-*glmS* reveals that GAP45 and β-tubulin are still located at the periphery of the knockdown parasites, suggesting that the initial stages of formation and attachment of the microtubules to the IMC are not affected (S6E Fig). SBF-SEM imaging confirmed the presence of the IMC, however the width of the plates was less than in the untreated controls, suggesting an inability of the IMC plates to expand (Fig 5F). A striking feature of the PhIL1-HA-*glmS* knockdown parasites was a greatly enlarged digestive vacuole (Fig 5F). While the parasite volume remained roughly similar (untreated, 45.2 ± 6.1 fL; treated, 51.4 ± 6 fL), the DV volume was markedly increased (Untreated, 1.9 ± 0.2 fL; treated, 10.8 ± 1.9 fL; P = 0.01) (Fig 5G). This may suggest mistrafficking of membrane destined for IMC expansion. Similarly, labeling live gametocytes with the pH-reporter, LysoSensor, reveals small, evenly distributed acidic compartments in control gametocytes and swollen acidic compartments in treated parasites (Fig 5H).

### PhIL1 co-precipitates with key IMC components

**Fig 6:**
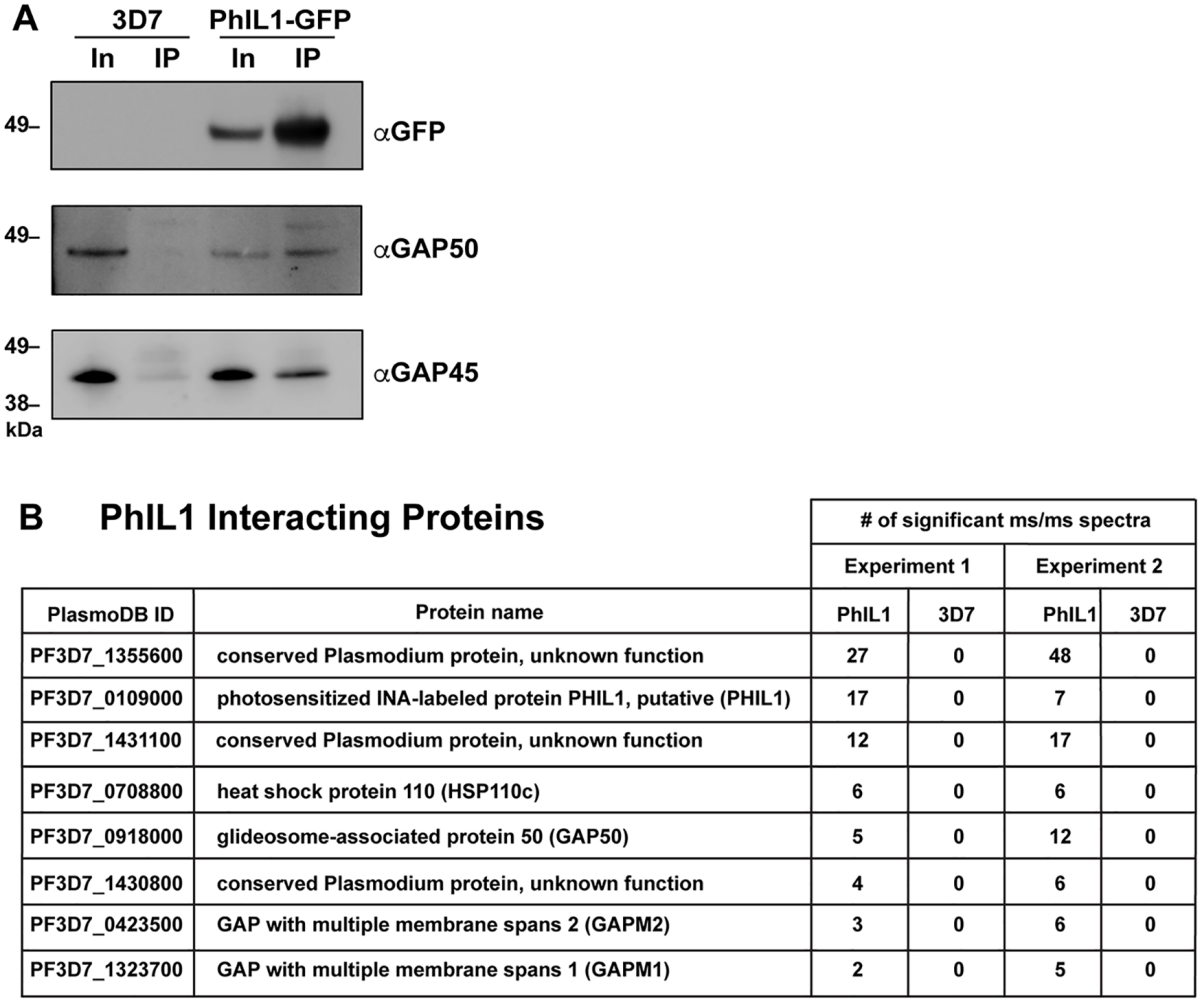
Identification of new IMC proteins in gametocytes. (A) 3D7 parent and PhIL1-GFP transfectant gametocytes, purified at stage IV, were detergent-solubilized and proteins were immunoprecipitated using GFP-Trap. The input and precipitated eluates (IP) were prepared for SDS-PAGE and Western blot and probed with anti-GFP, anti-GAP50 and anti-GAP45 antibodies. Molecular masses of markers are shown in kDa. (B) List of the PhIL1-interacting proteins. Two independent experiments were performed. Proteins that returned ≥2 significant MS/MS peptides in each experiment are included. A complete list of significant and non-significant proteins identified are presented in S2 Table.

To identify PhIL1-interacting proteins, we solubilized PhIL1-GFP stage IV gametocytes using Triton X-100 and performed immunoprecipitation using GFP-Trap. The IMC proteins, GAP45 and GAP50 were significantly enriched in the pull-down from the PhIL1-GFP transfectants, when compared to wildtype 3D7 (Fig 6A). To investigate PhIL1-interacting partners more globally, immunoprecipitated proteins were subjected to in-solution trypsin digestion and analysed by liquid chromatography-tandem mass spectrometry (LC-MS/MS). We identified eight parasite proteins that were significantly enriched (≥2 significant MS/MS spectra in two independent experiments) in PhIL1-GFP compared to wildtype 3D7 (Fig 6B, S2 Table). Precipitated proteins included PhIL1 itself, GAP50, two members of the glideosome-associated protein with multiple membrane spans (GAPM)1 and GAPM2, and Heat Shock Protein 110c (HSP110c) (Fig 6B, S2 Table). In addition, we identified three previously uncharacterized proteins PF3D7_1355600, PF3D7_1431100 and PF3D7_1430800, which we have termed **P**hIL1 **I**nteracting **P**roteins (PIPs)1-3 respectively (Fig 6B, S2 Table). PIP1 appears to be restricted to human malaria species with no homologue identified in the rodent malaria species, nor in *Toxoplasma*. Interrogation of transcriptional data (PlasmoDB) indicates that PIP1 is more highly transcribed in gametocytes than in asexual parasites [17]. In contrast, PIP2 and -3 are found in all malaria species, and exhibit low level similarity to the *T*. *gondii* alveolin domain-containing intermediate filament proteins, IMC7 and IMC12 (Fig 6B, S2 Table). PIP2 and PIP3 have been previously identified as putative IMC proteins [18]. Interactions with other known IMC and cytoskeleton proteins were also observed, including GAP40, GAP45, GAPM3, IMC1c, β-tubulin and actin-I, as well as with several uncharacterized proteins, but these failed to reach the applied significance criteria. A full list of identified proteins is provided in S2 Table.

### Characterization of PhIL1 interacting protein-1 (PIP1)

**Fig 7:**
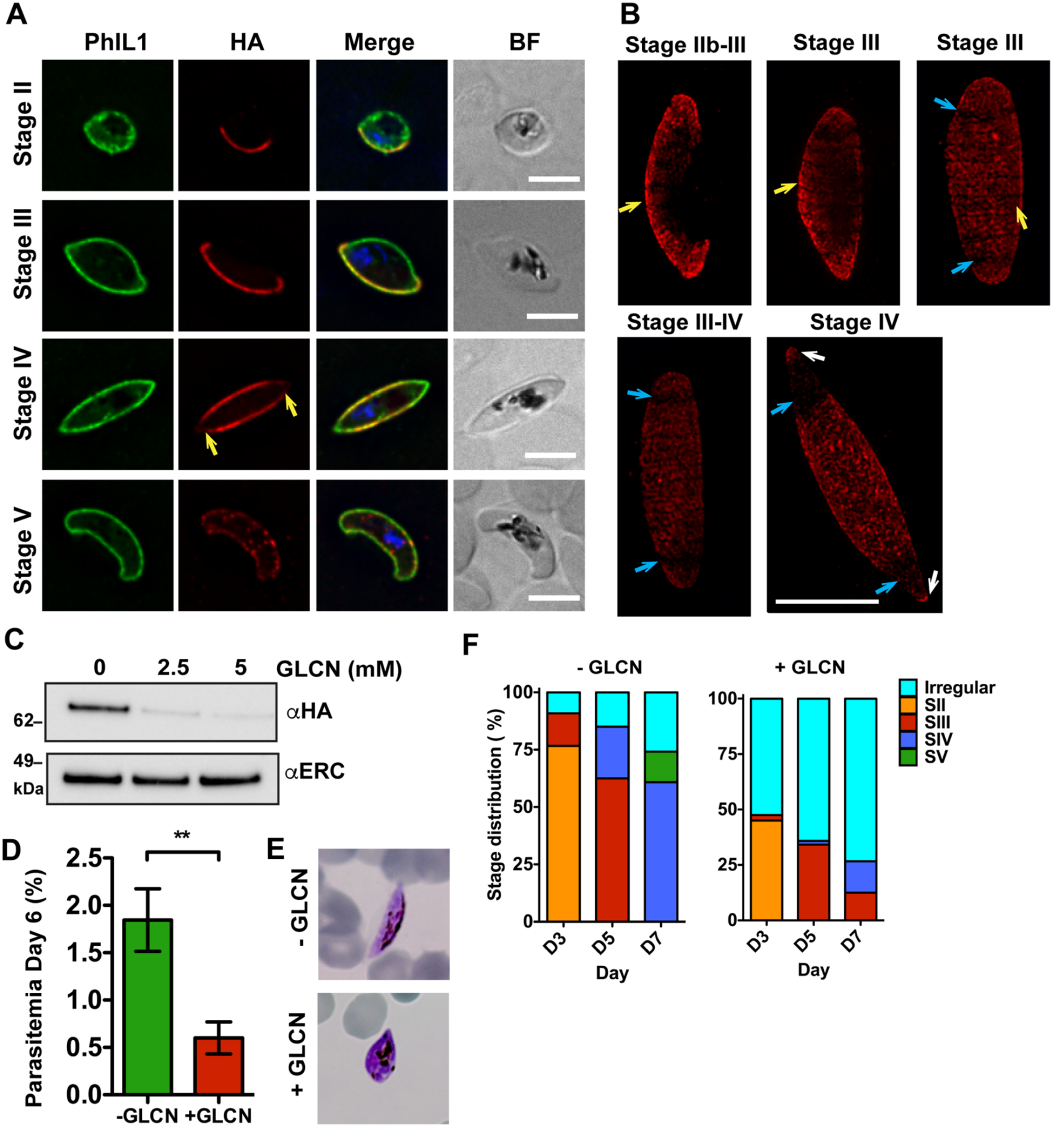
PhIL1-interacting protein, PIP1, is located at the gametocyte IMC and is essential for gametocyte development. (A) PIP1-HA-*glmS* transfectants were labeled with anti-PhIL1 rabbit (green) and anti-HA (red) at different developmental stages (II–V). Nuclei were labeled with DAPI. There is an apparent reduction in fluorescence intensity of PIP1-HA labeling at the tips of the gametocyte in stage IV (yellow arrow). In stage V, the fluorescence becomes punctate at the parasite periphery, while PhIL1 remains homogenous. (B) 3D-SIM imaging of PIP1-HA-*glmS* during gametocyte development. Homogeneous labeling of the IMC plates by anti-HA (red) are observed (yellow arrows) in the early stages of development. During stage III–IV of development, there is a loss of labeling from the plates near the tips of the elongated parasite (blue arrows), with some protein labeling of the tips themselves (white arrows). Scale bar: 5 μm. (C) Western blot analysis of saponin-treated pellets of harvested PIP1-HA-*glmS* plus or minus glucosamine. Probing with anti-HA antibodies shows a robust knockdown of PIP1-HA protein. (D) Parasitemia was calculated from Giemsa smears of PIP1-HA-*glmS* parasites plus or minus glucosamine. Data represent mean ± SEM. n = 3 experiments performed in triplicate. ** P <0.01, unpaired t-test. (E) Representative parasites from the Giemsa smears showing altered morphology following PIP1 knockdown. (F) Analysis of Giemsa smears from days 3, 5 and 7 showing a loss of morphologically normal parasites. The complete data set including wild type parasites plus or minus glucosamine are presented in S9C and S9D Fig. Data from 2 separate experiments performed in triplicate. Mean values are shown.

We generated a parasite line expressing a HA-*glmS* fusion of the top PhIL1 interacting protein (PF3D7_1355600; PIP1). Integration was confirmed by PCR (S8A and S8B Fig) and the expected band of ~66 kDa was observed in Western blots of both asexual and sexual stage parasites (S8D Fig). Immunofluorescence microscopy revealed that PIP1-HA is present at the IMC during early stages of development, where it largely co-locates with PhIL1 (Fig 7A). PIP1-HA extends completely around the parasite periphery by stage III of development (Fig 7A, top panels), but following transition to stage IV it is depleted from the tips of the gametocyte (Fig 7A, yellow arrows), and wanes significantly in stage V gametocytes (Fig 7A, bottom panel). Immunofluorescence microscopy reveals that PIP1-HA is located at the periphery of the nascent merozoites within late stage schizonts, in a similar location to PhIL1 (S8C Fig).

3D-SIM confirmed that PIP1 is associated with IMC plates in stage II—IV gametocytes (Fig 7B, yellow arrows). However, the plates closest to the gametocytes tip lose PIP1 fluorescence from stage III (Fig 7B, blue arrows). Some PIP1 fluorescence is retained at the apex of the tips in stage IV gametocytes (Fig 7B, white arrows). This is in contrast to PhIL1, which remains associated with all of the IMC plates throughout development (Fig 3B and 3E).

To confirm the interaction of PIP1 with PhIL1 we immunoprecipitated extracts of wild type 3D7 and PIP1-HA-*glmS* parasites, using anti-HA agarose beads. The precipitated proteins were subjected to in-solution tryptic digestion and analysed by mass spectrometry. This analysis identified PhIL1 as the top interacting protein, thus confirming this interaction. PIP2 and GAPM2 were also enriched (≥2 significant MS/MS spectra in two independent experiments) (S9F Fig and S2 Table). Other proteins enriched in one of the two experiments include PIP3, GAP50, GAP45 and α-tubulin II (S2 Table).

Treatment of the PIP1-HA-*glmS* parasites with 5 mM glucosamine was associated with ~90% apparent knockdown (Fig 7C). Immunofluorescence microscopy confirms the loss of the PIP1-HA signal (S8E Fig). Knockdown was associated with a 68% reduction in gametocyte numbers on day 6 of the gametocyte assay (P = 0.01) (Fig 7D), and an apparent arrest of development (Fig 7E). While the late stage marker Pfs48/45 is produced, less than 10% of the surviving PIP1-depleted parasites achieved stage IV morphology by day 7, compared with >50% of untreated parasites (Fig 7F, S9 Fig). The average parasite length decreased from 10.5 ± 0.5 μm to 5.2 ± 0.4 μm in the glucosamine-treated group (S9C Fig). As with PhIL1, electron microscopy revealed arrested development of the IMC and swelling of the digestive vacuole in the knockdown cell line (S9D Fig). Quantification of the SBF-SEM images showed no significant difference in the glucosamine-treated (46.6 ± 1.8 fL) and untreated (42.3 ± 2.3 fL) parasite volumes but a significant increase in the digestive vacuole volume following glucosamine treatment (untreated 0.9 ± 0.2 fL; treated 8.5 ± 2 fL; P = 0.01) (S9E Fig). By contrast, treatment of the parental 3D7 line with 5 mM glucosamine had no effect on gametocyte development (S9A Fig). Similarly, treatment of asexual PIP1-HA-*glmS* parasites had no significant effect on parasite growth (S9G Fig), suggesting that PIP1 is not essential (or only needed at a low level) for asexual replication.

## Discussion

The IMC is a cisternal compartment that is assembled under the plasma membrane of *Plasmodium* parasites, in the invasive or motile merozoite, sporozoite and ookinete stages, and in non-invasive/motile gametocytes. An IMC is also present in other Apicomplexan parasites, such as *T*. *gondii*, and related structures (more generally referred to as alveolar sacs) are found in all groups of the *Alveolates* (which unites Apicomplexan parasites, dinoflagellate algae, and the ciliates) [18, 19]. The IMC plays a range of structural roles, including underpinning the shape and stability of cells, providing a scaffold during cell division and harboring the machinery for gliding motility and cell invasion [19]. While some components of the IMC have shared functions in different stages of Apicomplexan development [20, 21], it is clear that some proteins carry out stage- and species-specific functions.

In the invasive or motile stages of *Plasmodium*, and in *T*. *gondii*, the IMC and the structural subpellicular microtubules are connected to tubulin-based annuli that lie under the plasma membrane at the apex of the cell, called the polar rings [22–24]. The three apical polar rings serve as a microtubule organizing center, and the subpellicular microtubules radiate from there toward the basal end of the cell [22, 25–27]. In cells with an apical complex the IMC appears to be laid down from the apical end by the deposition of material originating from the ER, with gradual expansion of the IMC towards the basal end of the cell [28, 29].

**Fig 8:**
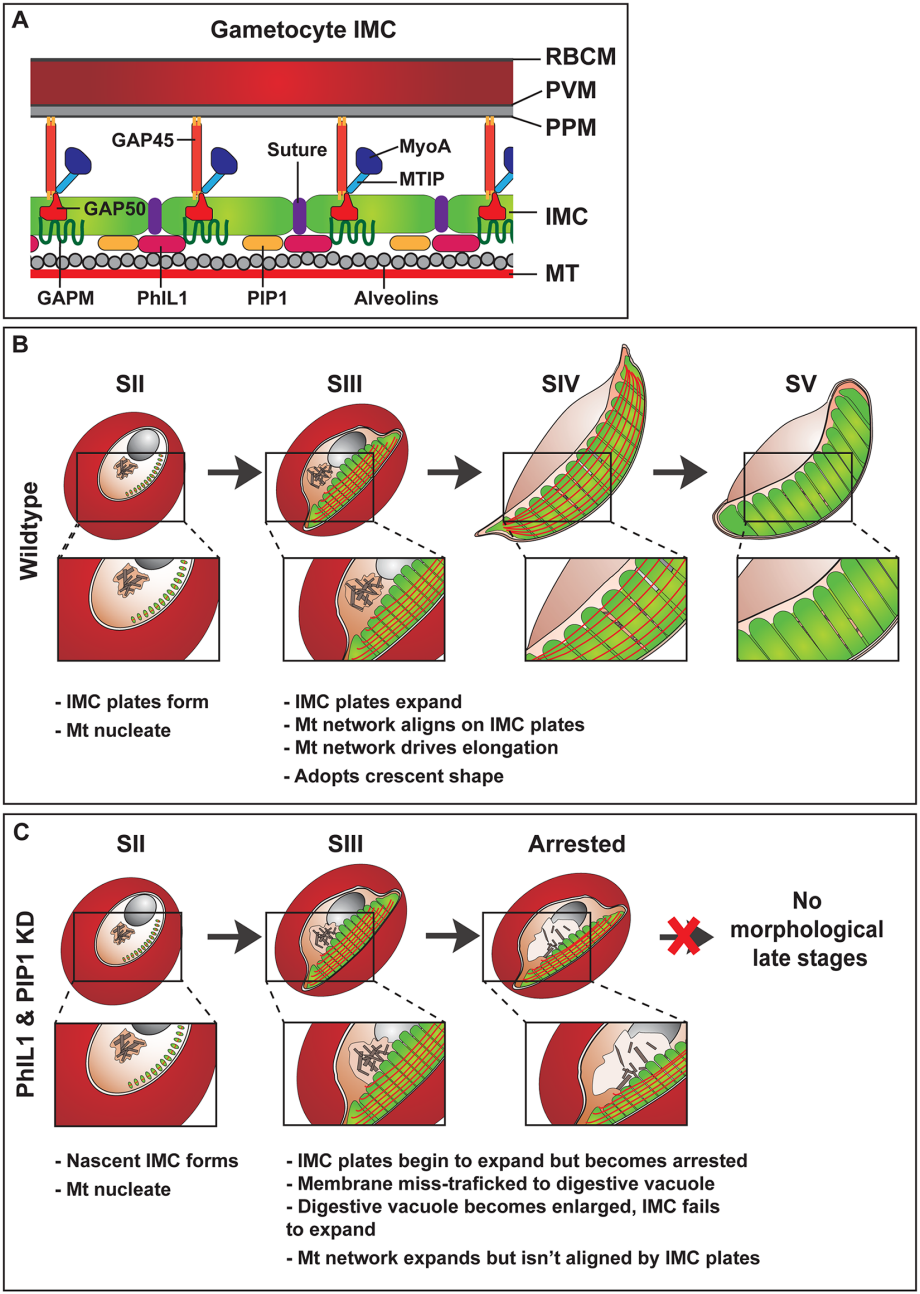
Model and schematic of the gametocyte IMC development. (A) Schematic showing the proposed positioning of proteins in the gametocyte IMC. Red blood cell membrane (RBCM); Parasitophorous vacuole membrane (PVM); parasite plasma membrane (PPM); inner membrane complex (IMC); microtubules (MT); Photosensitized 5-[125I] iodonaphthalene-1-azide labeled protein-1 (PhIL1); PhIL1-interacting protein 1 (PIP); Glideosome-associated protein 45 and 50 (GAP45 and 50); Glideosome-associated protein with multiple membrane spans (GAPM); myosin-A (MyoA); myosin-A tail domain interacting protein (MTIP). (B) Proposed model of plate formation and expansion during development and elongation of wild type gametocytes. IMC plates are deposited as 13 disk-like structures on the cytoplasmic side of the parasite plasma membrane. These plates act as a scaffold for microtubule formation. As the parasite develops, the plates expand through the addition of new membrane to the leading edges of the plates. The microtubule network aligns on these plates and drives parasite elongation. These plates continue to develop through to stage IV of development. At stage V, the microtubule network is disassembled but the IMC remains at the parasite periphery. (C) Gametocyte maturation in the absence of PhIL1 or PIP1. IMC plate formation is initiated as in wild type parasites, but new membrane is not added to the IMC membranes resulting in failure to organize the microtubule network and elongate the parasite. The membrane is miss-trafficked to the digestive vacuole leading to its swelling and enlargement. Disruption of PhIL1 and PIP1 leads to a significant reduction in parasite numbers and arrested morphological development.

By contrast, the gametocyte has no apical complex, and no obvious polarity, and we show here that the genesis of the IMC and the assembly of the underpinning microtubules occurs via a distinct mechanism (Fig 8). The initial event appears to be the deposition of IMC material in a thin semi-circular string of plates at the periphery of the roughly spherical stage I gametocyte. The nascent IMC appears to be segmented from the earliest stage, with individual segments acting to seed microtubule formation (Fig 8). Once established, the IMC expands laterally via the deposition of membrane at the leading edge, as evidenced by the thickened regions at the ends of the plates, observed in our SBF-SEM images. As the IMC tiles expand laterally, the microtubule stubs grow longitudinally, forming long filaments that appear to stretch the length of the cell (Fig 8). Microtubules also form in regions of the cell away from the IMC, but as the IMC expands laterally around the girth of the parasite, it associates with these microtubules and, as a consequence, the gametocyte constricts (laterally) and extends (longitudinally). By stage IV of development, the microtubules form a tight, sometimes slightly twisted array of longitudinally aligned microtubules around the entire, highly elongated parasite. The microtubules are depolymerized in stage V, but small membrane-bound stubs remain at the parasite periphery, generating an IMC-based carapace that retains a somewhat elongated shape, but is capable of deformation.

In this work, we characterized PhIL1 as a novel component of the gametocyte IMC and defined some of its interacting partners. PhIL1 has no homologues outside the phylum *Apicomplexa*. It is expressed at the periphery of the daughter merozoites in very late stage schizonts, consistent with being a component of the merozoite IMC. However the level of expression is low and the protein appears to be processed and knockdown does not affect asexual growth, indicating that it does not play a critical role in merogony or merozoite invasion. In cultures that have been induced to form gametocytes, PhIL1 is first observed at the periphery of the small membrane plates that initiate the IMC ribbon in stage I. As the plates expand, PhIL1 is distributed more uniformly across the plates, where it remains through to stage V of development. PhIL1 has no signal sequence and no transmembrane domain but appears to form a partially soluble (Triton X-100) protein complex with other components of the IMC as well as with cytoskeletal components, and may be lipid-modified, as reported for *T*. *gondii* PhIL1 [30]. Upon genetic knockdown of PhIL1, gametocytes form, but fail to develop morphologically beyond stage III and exhibit a swollen digestive vacuole and a significant loss of total gametocyte numbers. SBF-SEM imaging of wild type parasites shows an accumulation of extra membrane at the edges of the plates at the stage III-IV transition (Fig 8). It is possible that PhIL1 plays a role in recruiting membrane from the digestive vacuole to the IMC. The defect in elongation is similar to, though more dramatic than, that previously reported for *T*. *gondii* PhIL1. Genetic disruption of *Tg*PhIL1 resulted in tachyzoites that were significantly shorter and wider than wild type parasites, a phenotype that translated into a significant loss of virulence in a mouse model of the disease [31].

PhIL1 homologues are broadly represented within (but not outside) the Apicomplexa phylum. However only the C-terminal region of PhIL1 is conserved. The N-terminal region of *Pf*PhIL1 diverges from that of other Apicomplexan PhIL1 proteins, but is more well conserved amongst *Plasmodium* species. This region includes a domain that is recognized as a myosin and kinesin motor domain motif; however it appears to lack the P-loop motif characteristic of this family of NTPases. Further work is needed to determine the precise role of PhIL1, but a motor/tethering function could facilitate trafficking or docking of membrane vesicles to the IMC.

PIP1 was identified as the top-ranked PhIL1-interacting protein. PIP1 is rich in asparagine (15.8%) and lysine (13.2%) but contains no recognized motifs. Interestingly one of the top interacting proteins HSP110c has been previously shown to stabilize proteins containing asparagine repeat regions [32], and may play a role in maintaining correctly folded IMC and cytoskeleton proteins and trafficking cargo. While PhIL1 is an acidic protein (predicted pI = 4.81), PIP1 is highly basic (predicted pI = 9.68), which may contribute to their association. PIP1 is highly conserved across all *Plasmodium* species. Unlike PhIL1, PIP1 has no homologues outside the *Plasmodium* genus. Like PhIL1, PIP1 is present in the merozoite IMC, but does not appear to play an important role in asexual growth. In gametocytes, PIP1 is located in the IMC plates from their initiation to the final stages of development, but wanes later in development. As for PhIL1, knockdown of PIP1 expression was associated with a significant reduction in the number of gametocytes, and the surviving gametocytes fail to progress morphologically past stage II-III of development, indicating a critical role for PIP1 in IMC expansion (Fig 8).

In this work, we expanded the repertoire of potential gametocyte IMC proteins using immunoprecipitation to identify PhIL1-interacting proteins. Proteins that were significantly enriched in the PhIL1 immunoprecipitations include GAP50, GAPM-1 and -2, HSP110c and three new proteins termed PhIL1 interacting proteins (PIP)1-3. In addition, a number of known IMC proteins, including GAPM-2 and -3 and the cytoskeletal components, β-tubulin and actin-I, were identified but failed to reach significance due to the cut off level employed.

It is interesting to consider the roles of the different IMC components. The GAPM proteins (1–3) possess six transmembrane domains and are thought to be present in large, oligomeric complexes, potentially associated with 9 nm particles on the innermost (cytoplasmic) surface of the IMC [33, 34] and have been reported to interact with the alveolins [33]. The alveolins vary in size and contain multiple repeat domains with a motif that is predicted to form extended coiled-coil domains [21]. PIP2 and PIP3 share sequence similarity with the *Toxoplasma* proteins, IMC7 and IMC12, which contain alveolin domains. GAP50 is an integral membrane protein that is targeted via the endomembrane system to the IMC and is oriented with its N-terminal (non-functional) phosphatase domain facing the lumen of the IMC [28, 35]. GAP50 is thought to recruit pre-complexed GAP45-MTIP-MyoA on to the nascent IMC [28, 36]. It has been suggested that GAP45 spans the IMC and the plasma membranes, interacting with both, via myristyl and palmityl modification, thereby maintaining the integrity of the IMC during invasion [20]. Interactions with GAP45, GAP50 and PhIL1 may help stabilize the IMC, holding it close to the parasite plasma membrane as it expands and develops. *Tg*PhIL1 has been shown to be palmitylated [30] and this may provide a means of connecting the protein to the IMC. The nature of the interacting partners suggests that PhIL1 may be located on the cytoplasmic surface of the IMC and may participate in a higher order complex that links the microtubule-interacting proteins through to the structural components of the glideosome complex (Fig 8). This is supported by the solubility profile for PhIL1, which is consistent with a peripherally-located protein that is associated with the underlying actin/tubulin skeleton.

In summary, we have elucidated details of the genesis and elaboration of the gametocyte IMC and identified two novel gametocyte IMC proteins that appear to be involved in this process. This work gives new insights into the fascinating cell biology events that drive gametocyte elongation and facilitate preparation of the gametocyte for transfer to a mosquito host. Gametocyte formation and maturation represents a bottle-neck in parasite development and inhibition of this process would ablate disease transmission.

## Materials and methods

### Parasite culture

Asexual stage parasites were grown in O+ RBCs (Australian Red Cross blood service) at 5% hematocrit. The parasites were maintained in complete culture media containing RPMI-GlutaMAX-HEPES (Invitrogen) supplemented with 5% v/v human serum (Australian Red Cross blood service), 0.25% w/v AlbuMAX II (Invitrogen), 200 μM hypoxanthine, 10 mM D-glucose (Sigma) and 20 μg/ml gentamicin (Sigma). To obtain ring stage cultures sorbitol (5%) synchronization was performed [37]. Transfectants were maintained in media supplemented with WR99210 (5 nM). High gametocyte producing NF54 and 3D7 parasite lines were used [38, 39].

Gametocytes were generated as previously described [13, 40, 41]. Asexual stage parasites were sorbitol synchronized to produce a ring stage culture and the parasitemia adjusted to 3% parasitemia at 5% hematocrit (day -4). The culture was grown in complete culture media until day -1 resulting in a culture containing 8–10% trophozoite stage parasites. The culture was adjusted to 2% parasitemia 5% hematocrit by addition of fresh media and RBCs. The spent culture media was left on the culture at a ratio of 1:4 with fresh media to induce gametocyte induction. N-acetyl-D-glucosamine (GluNac; 62.5 mM final concentration) was added from day 0 of the culture [40]. This protocol results in synchronized gametocyte production. Gametocyte stage development was monitored by thin blood films and Giemsa staining. The following timing of stage progression is used as a guide but can vary depending on the cell line and age of asexual cells when the assay was established. Day 0–1: stage I; Day 2–3: stage II; Day 4–5: stage III, Day 6–7: stage IV; day 8 –onwards: stage V. Gametocytes were purified from culture at the required development stage by Percoll density gradient or magnet separation [40, 41].

### Plasmid construction and transfection

The *pPhIL1-3xHA* plasmid was created by amplifying full length *PhIL1* sequence from genomic 3D7 DNA with the P1FHA (GC**AGATCT**AAAATGCTTTCTTCCATATCACCAAAAAG) and P1RHA (AA**CTGCAG**CCATATCTTGGTTATAATTTTCTTGATC) primers (restriction enzyme sites in bold). The resulting PCR product was directionally cloned into the *Bgl*II and *Pst*I sites of the pD3HA plasmid. The *pPfs16-PhIL1-GFP* construct is an episomally maintained plasmid that utilizes the promoter region of the early gametocyte specific protein Pfs16 [12]. The full-length coding sequence of *PhIL1* was amplified from genomic 3D7 DNA with the PIGFPF (GC**GTCGAC**AAAATGCTTTCTTCCATATCACCAAAAAG) and PIGFPR (AA**CTGCAG**TGCTGCTGCTGCTGCTGCTGCTGCCATATCTTGGTTATAATTTTCTTGATC) primers containing *Bgl*II and *Pst*I restrictions sites respectively. This product was directionally cloned into the *p16proPfs16-GFP* Entry plasmid [12] which had been predigested with *Bgl*II and *Pst*I to remove the Pfs16 coding sequence. The resulting plasmid pPfs16pro-PhIL1-GFP was recombined with the *pHH1* Destination vector as previously described to obtain the *pHH1-Pfs16pro-PhIL1-GFP* plasmid [12].

The *pGLMS-PhIL1-HA* inducible knockdown construct was made as described above for *the pPhIL1-3xHA* plasmid, with cloning of the same full-length PhIL1 fragment into the *pGlmS* plasmid. To generate the *pGlmS-PIP1-HA* plasmids the coding sequence of *PIP1* was PCR amplified from 3D7 genomic DNA using the PIPF (GTCGACGGATCCATGAATAACGGATCTAATAAA) and PIPR (CTGCAGTGCTCTTTTTTTAAATTGCATAGG) primers and directionally cloned into the pGLMS-HA plasmid using the *Bam*HI and *Pst*I restriction sites.

Parasite transfections were performed by electroporation as previously described [42]. Drug cycling was performed to enrich for integrated parasites. Integration of the lines was confirmed by PCR using the PHILINT (GAACCTTCCATAACAGAC) or PIPINT (CCGCGGCCTATATTATTTTCATTAAACATTGAC) primers that target genomic sequence upstream of the targeting sequence in combination with the vector specific HArev (CGAACATTAAGCTGCCATAT) reverse primer.

### Recombinant protein and antibody production

A codon optimized (for *E*. *coli*) sequence of full length *PhIL1* (PlamsoDB ID: PF3D7_0109000.1) was commercially synthesized by GenScript. This sequence was directionally cloned into the *Pst*I and *Bgl*II sites of the pQE-30 protein expression plasmid, which will place a 6xHis tag at the N-terminus of the resulting protein. The plasmid was transformed into BL21 (DE3) *E*. *coli* (Bioline) and grown in the presence of 100 μg/ml Ampicillin. The expression of recombinant PhIL1 was induced with the addition of 2 mM isopropyl-D-1-thiogalactopyranosid (IPTG). After 3 h, cells were pelleted via centrifugation at 4,000 x g for 20 min at 4°C and the pellet was then re-suspended in lysis buffer (50 mM NaH_2_PO_4_, 300 mM NaCl, 10 mM imidazole, pH 8.0). Lysozyme (1 mg/mL) was also added and cells were incubated on ice for 30 min. The lysate was then sonicated and drawn through a 21-gauge syringe before being frozen at -80°C overnight. After thawing, the lysate was centrifuged at 10,000 x g for 20 min at 4°C and the supernatant containing the soluble protein fraction was discarded. The pellet was re-suspended in a denaturing lysis buffer (100 mM NaH_2_PO_4_, 10 mM Tris-Cl, 8 M urea, pH 8.0) and after a 1 h incubation period, the *E*. *coli* lysate was centrifuged at 10,000 x g for 20 min. His-tagged PhIL1 was purified from the supernatant fraction using a batch purification method.

The bacterial lysate was mixed with 50% Ni-NTA agarose resin for 1 h on a rotary mixer and then placed into a column. The agarose resin was washed three times using a wash buffer (100 mM NaH_2_PO_4_, 10 mM Tris-Cl, 8 M urea, pH 6.3). Recombinant PhIL1 was eluted using elution buffer A (100 mM NaH_2_PO_4_, 10 mM Tris-Cl, 8M urea, pH 5.9) and then elution buffer B (100 mM NaH_2_PO_4_, 10 mM Tris-Cl, 8 M urea, pH 4.5). All elution fractions were collected. Those containing PhIL1 protein were pooled and the buffer was exchanged for 1x PBS via dialysis. The resultant protein was then sent to the Walter and Eliza Hall Institute antibody facility, Bundoora. Rabbits were immunized to produce polyclonal antibodies against *P*. *falciparum* PhIL1.

### Immunofluorescence microscopy

Glass coverslips were primed for immunofluorescence assays by incubating with 0.1 mg/mL PHAE (erythroagglutinating phytohemagglutinin, Sigma Aldrich) for 15–30 min in a humid chamber at 37°C [43]. Parasites were harvested by centrifugation at 2,000 x g for 2 min, washed in 1x PBS and placed onto the PHAE coated coverslip and incubated at room temperature for 15 min. Washing of samples with 1x PBS was carried out between each step of the following protocol. Cells were incubated on the slides and then fixed in a solution of 4% v/v paraformaldehyde and 0.0065% v/v glutaraldehyde. Parasite permeabilization was carried out using 0.1% Triton X-100. Immobilized cells were stained with primary antibodies for 1 h at room temperature (RT) (anti-PhIL1 rabbit (1:500), anti-HA rat (1:250, Sigma Aldrich) anti-HA mouse (1:250, Sigma Aldrich), anti-GAP45 rabbit (1:1000, [44]) anti-GFP mouse (1:500, Roche) anti-β-tubulin mouse (1:500, Sigma Aldrich), anti-Pfs16 mouse (1:1000, [12]). The secondary antibody was added for 1 h at RT (goat anti-mouse Alexa Fluor 488, 568, 647; anti-rabbit 488, 568, 647; anti-rat 647, were used at 1:250). Parasite nuclei were stained with DAPI (2 μg/mL) for 10 min at room temperature. The slides were mounted in *p*-phenylenediamine antifade and kept at 4°C. Samples were imaged on a DeltaVision Elite Restorative Widefield Deconvolution Imaging System (GE Healthcare) using the 100x UPLS Apo (1.4NA) objective lens under oil immersion. Samples were excited with solid state illumination (Insight SSI, Lumencor). The following filter sets with excitation and emission wavelengths were used: DAPI Ex390/18, Em435/48; FITC, Ex475/28, Em523/26; TRITC, Ex542/27, Em594/45; Cy5 Ex 632/22, 676/34 nm. For higher resolution imaging via 3D Structured Illumination microscopy (3D-SIM), the DeltaVision OMX V4 Blaze was used (GE Healthcare). Samples were excited using 488, 568 or 642 nm lasers and imaged using band pass filters at 528/48, 609/37 and 683/40 nm with a 60X Olympus Plan APO N (1.42 NA) oil immersion lens. Images were processed using the Fiji ImageJ software [45].

### Solubility studies

For the solubility assay, a culture enriched in gametocytes from stage III to stage V was magnet purified. Gametocytes were lysed in 0.03% saponin and the pellet fractions incubated with 0.1 M sodium carbonate, pH 11 (4°C) or in 8 M urea (RT) or 1% Triton X-100 in PBS, pH 7.4 (4°C) or in 2% SDS (RT) for 30 min. The soluble supernatant and the insoluble pellet were collected and taken up in 1x SDS loading buffer. Proteins fractions were analysed by immunoblotting.

### Immunoblotting

For SDS-PAGE, protein samples were prepared by lysis with 0.03% saponin for 25 min on ice. Protein samples were separated on 4–12% Bolt Bis-Tris gels (Invitrogen) and transferred to 0.2 μm nitrocellulose membrane using the iBlot system (Invitrogen). The nitrocellulose membrane was blocked in 3.5% skim milk/1xPBS for 1 h at RT prior to antibody probing. Primary and secondary antibodies were prepared in 3.5% skim milk and incubated with the membranes for 1 h at RT. The membranes were washed with 1x PBS/0.05% Tween20 after the primary and secondary antibody incubations. The following primary antibodies were used in this study: anti-PhIL1 pre/post-immune (1:500), anti-HA mouse (1:500, Sigma Aldrich), anti-ERC (1:1000), anti-GAP45 (1:1000, [44]), anti-GAP50 (1:500, [44], anti-GFP, 1:500, Roche)). Horseradish peroxidase conjugated anti-mouse or anti-rabbit antibodies were used (1:25,000, Millipore). The washed immunoblots were incubated with enhanced chemiluminescent (ECL) reagents before being imaged using the FujiFilm LAS3000 digital imaging system (GE Healthcare).

### Electron microscopy-high pressure freezing/Freeze substitution

Gametocytes were fixed using 2% paraformaldehyde and 0.1% glutaraldehyde. Cells were mounted to membrane carriers (Leica Microsystems) with a cell depth of 200 μm. The cells were then rapidly frozen by liquid nitrogen jet under 2100 bar pressure in a high pressure freezer (EM PACT2, Leica Microsystems) and quickly transferred to liquid nitrogen. A quick freeze substitution method was used (McDonald and Webb, 2011). The samples were transferred to cryotubes containing fixative: 1% osmium tetroxide, 0.5% uranyl acetate 5% water in acetone at liquid nitrogen temperature. The cryotubes were mounted on a cooled aluminum heat block in a polystyrene box filled with liquid nitrogen. To initiate the freeze substitution, the liquid nitrogen was replaced with dry ice pellets. The box was placed on a rotary shaker at 125 rpm. The dry ice was removed after 2 h and the shaking continued. After 1 h the cryotubes were removed from the block.

### Transmission electron microscopy

The samples were washed three times with pure acetone after returning to RT. They were then infiltrated and embedded with EPON resin. Polymerization of the resin was carried out at 60°C before 60 nm sections were prepared with an ultramicrotome (Leica EM UC7, Leica Microsystems). The specimens were post-stained with 7% uranyl acetate in methanol and Reynold’s lead citrate. Samples were observed on Transmission Electron Microscope (TEM) at 200 kV (Tecnai G2 F30, FEI).

### Serial block face-scanning electron microscopy (SBF-SEM)

The staining method (ROTO) was adapted from a previous publication [46]. Parasite pellets were fixed with 2.5% glutaraldehyde in PBS for 1 h at 4°C. Following agarose pre-embedding and washing with 0.175 M sodium cacodylate buffer, cells were stained in ferrocyanide-reduced osmium tetroxide in 0.1 M cacodylate buffer for 1 h on ice. After washing with double distilled H_2_O, the cells were incubated with freshly prepared 1% thiocarbonhydrazide solution in H_2_O for 20 min at RT. The cells were then rinsed and further stained with 2% osmium tetroxide in H_2_O for 30 min at RT. Subsequently the cells were rinsed and *en-bloc* stained with 1% uranyl acetate in H_2_O overnight at RT. In the final step of staining the specimens were rinsed and treated *en-bloc* with Walton’s lead aspartate for 30 min at 60°C [47]. Following the removal of lead aspartate and several washes, the cells were dehydrated in a graded series of ethanol-H_2_O mixes, followed by several washes in dry ethanol and dry acetone and then progressively infiltrated with EPON resin. After resin polymerization, a 200 x 200 x 200 μm resin block was trimmed off using the mesa trimming method [48] on an ultramicrotome (Leica EM UC7, Leica Microsystems). The resin block was mounted on a microtome stub and immobilized using silver glue. After the silver glue was completely dried, the resin block was coated with a layer of gold and the top surface was then cleansed by diamond knife. The serial images (every 50 nm) were collected by a serial block face-scanning electron microscope (SEM), which was equipped with an in-chamber diamond knife, (Teneo VolumeScope, FEI Company) using back scattered electron signals at 3 kV under low vacuum conditions.

### SBF-SEM image analysis

Serial sections were optimized and aligned using IMOD software (Boulder Laboratory for 3D Electron Microscopy of Cells). The regions of interest were segmented and reconstructed into 3D models, using a semi-automatic method described previously [49]. Briefly, the pixel size of the greyscale images was binned to 20–50 nm, the images were subjected to Gaussian smoothing and a threshold value was selected manually from each reconstructed tomogram using a noise-estimate variance criterion, after marking some of the relevant cellular features, as described by others [50]. Connected regions were identified semi-automatically using connected component analysis [51]. Where required, a rough bounding area was drawn manually on every a few 2D sections. The regions were labeled with different colors, and 3D models were generated and rendered [52]. The volumes of the 3D models were determined for the organelles, while the parasite volume was determined after manual segmentation.

For polarity assessment, the IMOD Slicer tool was used to orient slices at the maximum length or width of the cells and organelles and the relative distances from the ends were measured using open contours in IMOD. The correct positioning of the open contours was confirmed on 3D models.

### Immunoprecipitation and mass spectrometry

Purified gametocytes were harvested and washed in 1xPBS for co-immunoprecipitation experiments. Parasites were solubilized using 1% TX-100/PBS (in 150 mM NaCl, 50 mM Tris, 2 mM EDTA) plus Complete protease inhibitors (Roche) on ice for 30 min, re-suspending every 10 min. Insoluble material was pelleted by centrifugation 16,000 x g for 10 min at 4°C. The supernatant was also re-pelleted to remove any residual insoluble material. The soluble fraction was incubated with 50 μL of Pierce protein A agarose beads (Pierce) for 30 min at 4°C to remove non-specific binding proteins. The resin was pelleted by centrifugation at 3,420 x g for 2 min and supernatant was incubated for 2 h with 25 μL GFP-Trap A (Chromotek) or anti-HA agarose (Sigma) depending on parasite cell line, at 4°C. After pelleting the resin at 3,420 g for 2 min, the beads were washed five times with 1% Triton X-100. Proteins were then either eluted using SDS and analysed via Western blotting or the beads were washed two times with 1 mM Tris and analysed via mass spectrometry.

For mass spectrometry, proteins was eluted from beads with 5 μL trifluroethanol and 20 μL 0.1% formic acid (pH 2.5) and incubated at 50°C for 5 min. Beads were pelleted at 3,420 x g for 2 min and 20 μL of the supernatant collected. The sample was neutralized with the addition of 1 μL of 1 M tetraethylammonium bicarbonate. To reduce disulphide bonds, 0.2 μL of tris(2-carboxyethyl)phosphine (final concentration of 5 mM) was added to the supernatant and the solution was incubated at 70°C for 10 min. Proteins were digested with trypsin overnight at 37°C. Mass spectrometry was performed using a Thermo NanoLC/ OrbiTRAP ELITE ETD for GFP-Trap samples, and a Thermo NanoLC/ Q Exactive Plus for the anti-HA Agarose samples. Mass spectra were searched in MASCOT (Matrix Science) against a custom database comprised of *P*. *falciparum* (PlasmoDB) and *H*. *sapiens* (UniProt) non-redundant proteomes. Protein hits were considered significant if they were present in two biological replicates, had ≥2 significant peptide MS/MS spectra, and were at least 5 times enriched compared to the 3D7 or NF54 parental control. The mass spectrometry proteomics data have been deposited to the ProteomeXchange Consortium via the PRIDE [53] partner repository with the dataset identifier PXD007564.

### Quantification and statistical analysis

All statistical analyses were performed using the Graph Pad Prism 5 software package. P values were calculated using an unpaired t-test, the mean values plus or minus the standard error are shown. This information is provided in the figure legends. The numbers of repeat experiments, internal replicates and cell numbers measured are noted in the figure legends.

### Ethics statement

Red Blood Cells and serum was obtained from the Australian Red Cross blood service. All blood products were anonymous and individual donors could not be identified. This work was approved by the University of Melbourne Human Research Ethics Committee (Approval number 1135799).

## Supporting information

S1 Fig Quantitative analysis of cellular compartment dimensions. **Related to** Fig 1. Length and width measurements for (A) parasite, (B) nucleus and (C) mitochondrion assessed from SBF-SEM data for stage II–V gametocytes. Data represent mean ± SEM. The Tables below the graphs show the number of cells assessed, mean values, standard deviations and the standard errors for each set of measurements. Unpaired t-test; * P <0.1; ** P <0.01; *** P <0.001; **** P <0.0001. Only significant differences are shown all other differences are non significant.
(TIF)

S2 Fig Relative organelle position at different stages of gametocyte development. **Related to** Fig 1. Average lengths and heights for the parasite (blue), the nucleus (yellow) and the mitochondrion (red) at stage II (A), III (B), IV (C) and V (D). The individual measurements are provided in S1 Table.
(TIF)

S3 Fig Sequence alignments of PhIL1 homologues. **Related to** Fig 3. Sequence alignment of PhIL1 homologs in Apicomplexan parasites. Sequences were obtained from the EUPath website and a multiple sequence alignment of the annotated protein sequences was performed using Clustal Omega. The following sequences were used: PF3D7_0109000, *P*. *falciparum* 3D7, photosensitized INA-labeled protein PHIL1, putative; PVX_081335, *P*. *vivax* Sal-1, hypothetical protein, conserved; PBANKA_0204600, *P*. *berghei* ANKA, photosensitized INA-labeled protein PHIL1, putative; HA_258410, *Hammondia hammondi* strain H.H.34, photosensitized INA-labeled protein PHIL1; TGME49_258410, *T*. *gondii* ME49, photosensitized INA-labeled protein PHIL1; cyc_07347, *Cyclospora cayetanensis* strain CHN_HEN01, photosensitized ina-labeled protein phil1; NCLIV_028090, *Neospora caninum* Liverpool, conserved hypothetical protein; ETH_00020575, *Eimeria tenella* strain Houghton, PhIL1, related protein.
(TIF)

S4 Fig PhIL1 is located at the IMC in schizonts and gametocytes. **Related to** Fig 3. (A,B) Immunofluorescence microscopy of schizont-stage asexual parasites (PhIL1-HA), labeled with rabbit anti-PhIL1 (green), mouse anti-HA (red) and rabbit anti-GAP45 (green). Nuclei were stained with DAPI. Scale bar: 5 μm. (C) Western blot analysis of saponin-treated pellets of stage IV gametocytes harvested from PhIL1-GFP and PhIL1-HA transfectants. The membrane was probed with anti-PhIL1, anti-HA, anti-GFP, anti-GAP45 and anti-ERC (as a loading control). Right hand side: Schizont extracts were probed with anti-PhIL1 antiserum.
(TIF)

S5 Fig Freeze substitution reveals native microtubule organization. **Related to** Fig 4. (A) Electron micrographs of stage II gametocytes showing microtubules (yellow arrow) lying along the developing IMC (red arrows). (B) Stage II gametocyte cut longitudinally highlighting microtubules crossing the cytoplasm (blue arrows) and IMC-associated microtubules (yellow arrows). The IMC has developed ahead of the microtubule network. (C) Cross-sectional and (D) longitudinal views of a stage III gametocyte showing microtubules (yellow arrows) underneath the IMC. (E-F) Longitudinal sections highlighting the microtubules associated with the IMC along the body of the gametocyte (blue arrow) and the arrangement of the microtubule network at the tips of the gametocyte (yellow arrow). (G-H) Stage V gametocytes, showing cross-sectional (G) and longitudinal (H) views highlighting the disassembled microtubule network and the remnant stubs of microtubules at the parasite periphery (yellow arrows). The RBC membrane, PVM, and the double membrane of the IMC are indicated. Scale bars: 200 nm.
(TIF)

S6 Fig Characterization of PhIL1-HA-*glmS* parasites. **Related to** Fig 5. (A) Schematic of the *Phil1* genomic locus, the vector and the integrated vector showing primer placement. (B) PCR-based confirmation of integration of *pPhIL1-HA*-*glmS* into the genomic locus. Gametocyte stage progression counts from Giemsa smears taken on days 2–7 of gametocyte development for wild type NF54 (C) and PhIL1-HA-*glmS* (D) parasites, plus or minus 5 mM glucosamine. The percentage of each parasite stage on each day is presented. The data represent the average of 3 separate experiments. Stages are color-coded and “irregular” refers to parasites that do not fit morphologically into a gametocyte stage description. (E) Immunofluorescence microscopy confirming the presence of the IMC, labeled with anti-GAP45 (green) and microtubules, labeled with anti-β-tubulin (red), at the parasite periphery following PhIL1 knockdown. Scale bar: 5 μm. (F) Average cell length measurements for wild type NF54 and PhIL1-HA-*glmS* parasites plus or minus glucosamine. Data are represented as mean ± SEM; n = 11 cells.
(TIF)

S7 Fig Asexual and sexual development of PhIL1-HA-*glmS* parasites. **Related to** Fig 5. (A) Counts of wild type NF54 and PhIL1-HA-*glmS* asexual parasites subjected to a range of glucosamine concentrations. Parasites were treated for 48 hours with or without glucosamine from the ring stage. The data represent the mean ± SEM for 3 separate experiments. (B) Immunofluorescence analysis of PhIL1-HA-glmS parasite at day 8 of development in treated and untreated conditions. The images reveal the presence of Pf48/45 (green) at the parasite periphery following knockdown. Staining with anti-GAP45 (red) shows that the IMC is present in both the treated and untreated samples. Nuclei are stained with DAPI (blue). Scale bar: 5 μm.
(TIF)

S8 Fig Analysis of PIP1-HA-*glmS* parasites. **Related to** Fig 7. (A) Schematic of the *PIP1* genomic locus, the vector and the integrated vector showing primer placement. (B) PCR-based confirmation of integration of *pPIP-HA*-*glmS* into the genomic locus. (C) Schizonts were prepared for immunofluorescence microscopy and probed with anti-HA (red) and anti-PhIL1 (green), revealing the presence of PIP1 at the IMC. Nuclei are stained with DAPI. Scale bars: 5 μm. (D) Western analysis of PIP1-HA-*glmS* extracts from schizont and stage IV gametocytes, probed with anti-HA, revealing a protein of the expected size for HA tagged PIP1. ERC is probed as a loading control. (E) Immunofluorescence analysis of PIP1-HA-*glmS* parasite at day 6 of development in treated and untreated conditions. The images illustrate the maintenance of PhIL1 (green) at the parasite periphery following knockdown and the significant reduction of HA (red) labeling in the glucosamine-treated samples. Nuclei are stained with DAPI. Scale bar: 5 μm. (F) Immunofluorescence analysis of PIP1-HA-*glmS* parasite at day 8 of development, showing Pf48/45 (green) at the parasite periphery following knockdown (and in controls). Staining with anti-GAP45 (red) shows that the IMC is present at the cell periphery in both samples. Nuclei are stained with DAPI (blue). Scale bar: 5 μm.
(TIF)

S9 Fig Characterization of PIP1-HA-*glmS* parasites. **Related to** Fig 7. Giemsa-stained parasite smears were used to assess development over days 2 to 7 following induction of gametocytogenesis. (A) Control 3D7 parasites were compared to (B) PIP1-HA-*glmS* transfectants following induction and addition of 5 mM glucosamine. (C) The length of gametocytes was assessed on day 6 after induction. Data are represented as mean ± SEM; n = 15 cells. (D) Transmission electron microscopy (TEM) thin sections (50 nm) of PIP1-HA-*glmS* parasites plus or minus glucosamine. The IMC is observed at the periphery of the parasite (yellow arrow). The digestive vacuole (DV) is swollen when PIP1 is knocked down (blue arrows). The IMC and RBC membranes are labeled. Scale bar: 1 μm. (E) Quantification of the SBF-SEM images. Mean volumes for the parasite and the digestive vacuole are shown. Data represent mean ± SEM. n = 5. ** P <0.01, unpaired t-test. (F) PIP1 interacting proteins. Two independent experiments were performed. Proteins that returned ≥2 significant MS/MS peptides in each experiment are included. A complete list of significant and non-significant proteins identified can be found in S2 Table. (G) Counts of wild type 3D7 and PIP1-HA-*glmS* asexual parasites subjected to a range of glucosamine concentrations. Parasites were treated for 48 hours with or without glucosamine from ring stage. The data represent the mean ± SEM for 3 separate experiments.
(TIF)

S10 Fig Full length Western blots. **Related to** Fig 3. Western blot analysis of saponin-treated pellets of 3D7 and GAP50-GFP stage IV gametocytes. Gametocytes were probed with PhIL1 pre-immune serum, anti-PhIL1 antiserum, anti-GAP45, anti-GFP and anti-ERC.
(TIF)

S1 Video Translations through the SBF-SEM virtual sections of stage I, II and IIa gametocytes. **Related to** Fig 1. These translations correspond to the images shown in Fig 1. Organelles are marked in the sections displayed in Figs 1 and 4.
(AVI)

S2 Video Translations through the SBF-SEM virtual sections of stage IIb and IIIa gametocytes. **Related to** Fig 1. These translations correspond to the images shown in Fig 1. Organelles are marked in the sections displayed in Figs 1 and 4.
(AVI)

S3 Video Translations through the SBF-SEM virtual sections of stage IIIb, IV and V gametocytes. **Related to** Fig 1. These translations correspond to the images shown in Fig 1. Organelles are marked in the sections displayed in Figs 1 and 4.
(AVI)

S4 Video SBF-SEM whole cell reconstructions of stage I, II and IIa gametocytes. **Related to** Fig 1. Rendered models of SBF-SEM imaged cells. The following structures are labeled in the sections (top) and color-coded in the rendered models (bottom). Parasite plasma membrane/parasitophorous vacuole membrane (PM, blue), inner membrane complex (IMC, purple), mitochondrion (M, red), nucleus (N, yellow), endoplasmic reticulum (ER), hemozoin (Hz), the apicoplast (A, orange), osmophillic bodies (OB, light blue) and RBC (maroon). These rotations correspond to the images shown in Fig 1.
(AVI)

S5 Video SBF-SEM whole cell reconstructions of stage IIb and IIIa gametocytes. **Related to** Fig 1. Rendered models of SBF-SEM imaged cells. The following structures are labeled in the sections (top) and color-coded in the rendered models (bottom). Parasite plasma membrane/parasitophorous vacuole membrane (PM, blue), inner membrane complex (IMC, purple), mitochondrion (M, red), nucleus (N, yellow), endoplasmic reticulum (ER), hemozoin (Hz), the apicoplast (A, orange), osmophillic bodies (OB, light blue) and RBC (maroon). These rotations correspond to the images shown in Fig 1.
(AVI)

S6 Video SBF-SEM whole cell reconstructions of stage IIIb, IV and V gametocytes. **Related to** Fig 1. Rendered models of SBF-SEM imaged cells. The following structures are labeled in the sections (top) and color-coded in the rendered models (bottom). Parasite plasma membrane/parasitophorous vacuole membrane (PM, blue), inner membrane complex (IMC, purple), mitochondrion (M, red), nucleus (N, yellow), endoplasmic reticulum (ER), hemozoin (Hz), the apicoplast (A, orange), osmophillic bodies (OB, light blue) and RBC (maroon). These rotations correspond to the images shown in Fig 1.
(AVI)

S7 Video Rotations of 3D-SIM images of stage I, II and IIb gametocytes. **Related to** Fig 4. 3D rotations showing the location of PhIL1-HA (red) and β-tubulin (green) labeled microtubules, across stage I-IIb of development.
(AVI)

S8 Video Rotations of 3D-SIM images of Stage III, IV and V gametocytes. **Related to** Fig 4. 3D rotations showing the location of PhIL1-HA (red) and β-tubulin (green) labeled microtubules, across stage III-V of development.
(AVI)

S1 Table Relative organelle position of individual cells. **Related to** Fig 1 **and** S1 and S2 Figs. Length and Width measurements of the SBF-SEM data for Stage II–V gametocytes and the relative positions of the organelles are shown for each of the 10 cells measured. Each stage is separated into their own tabs in the excel spreadsheet.
(XLSX)

S2 Table Mass spectrometry search data and analysis of PHIL1-GFP and PIP1-HA co-immunoprecipitation experiments. **Related to** Fig 6 **and** S9 Fig. Each tab within this excel spread sheet is clearly labeled with the experiment and the results displayed within. PHIL1-GFP, PIP1-HA and 3D7 control data from *H*. *sapiens* and *P*. *falciparum* MASCOT searches. Further annotations are provided within the data. Proteins were considered enriched if they were only present in the PHIL1-GFP samples, or were 5-fold enriched compared to 3D7.
(XLSX)
