# Supplementary figures and images for: Disrupting assembly of the inner membrane complex blocks Plasmodium falciparum sexual stage development
Source: PLoS Pathog. 2017 Oct 6;13(10):e1006659. doi: 10.1371/journal.ppat.1006659 (PMC5646874; doi:10.1371/journal.ppat.1006659)

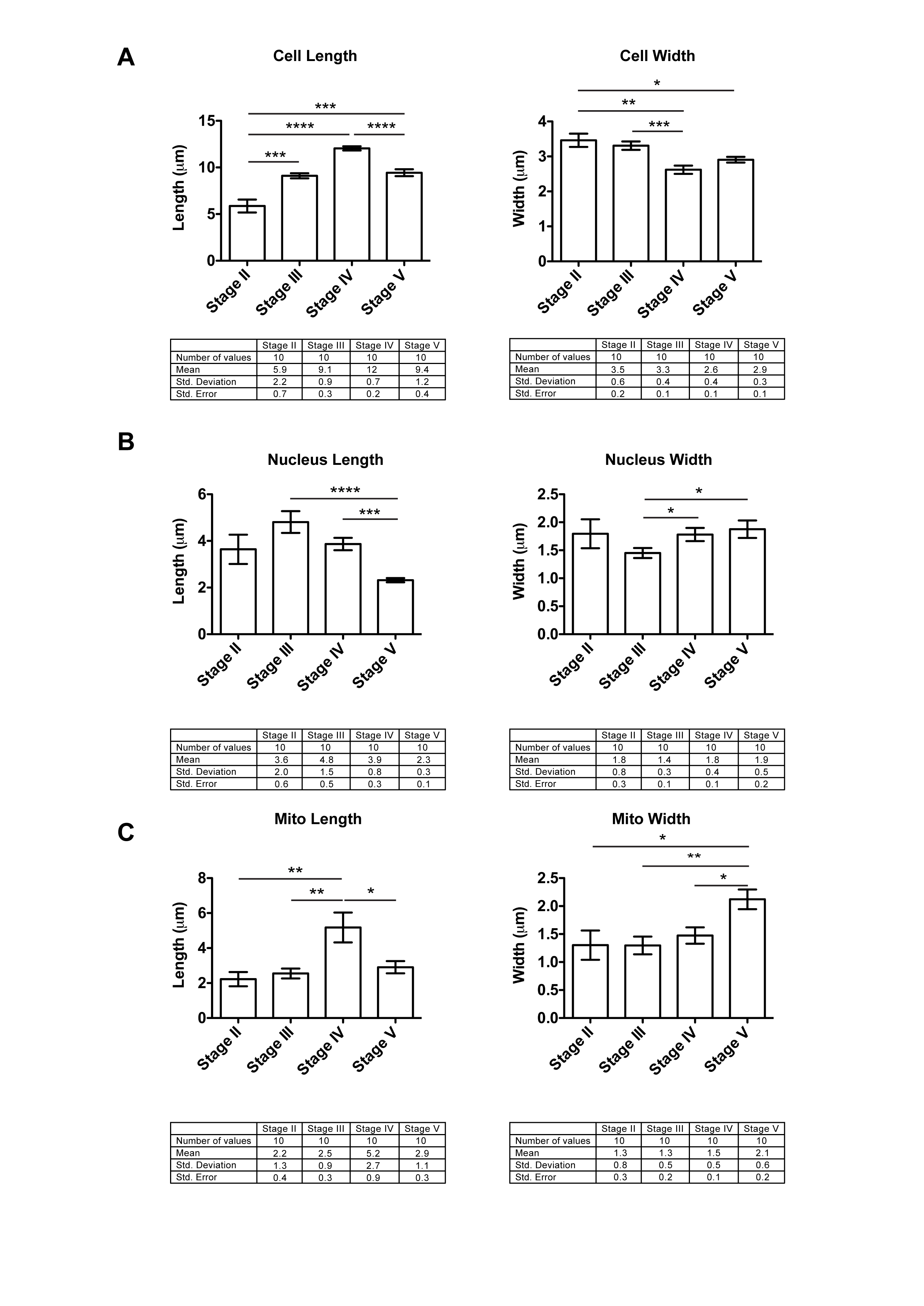

Supplement: S1 Fig — Related to Fig 1. Length and width measurements for (A) parasite, (B) nucleus and (C) mitochondrion assessed from SBF-SEM data for stage II–V gametocytes. Data represent mean ± SEM. The Tables below the graphs show the number of cells assessed, mean values, standard deviations and the standard errors for each set of measurements. Unpaired t-test; * P <0.1; ** P <0.01; *** P <0.001; **** P <0.0001. Only significant differences are shown all other differences are non significant. (TIF) [file ppat.1006659.s001.tif]

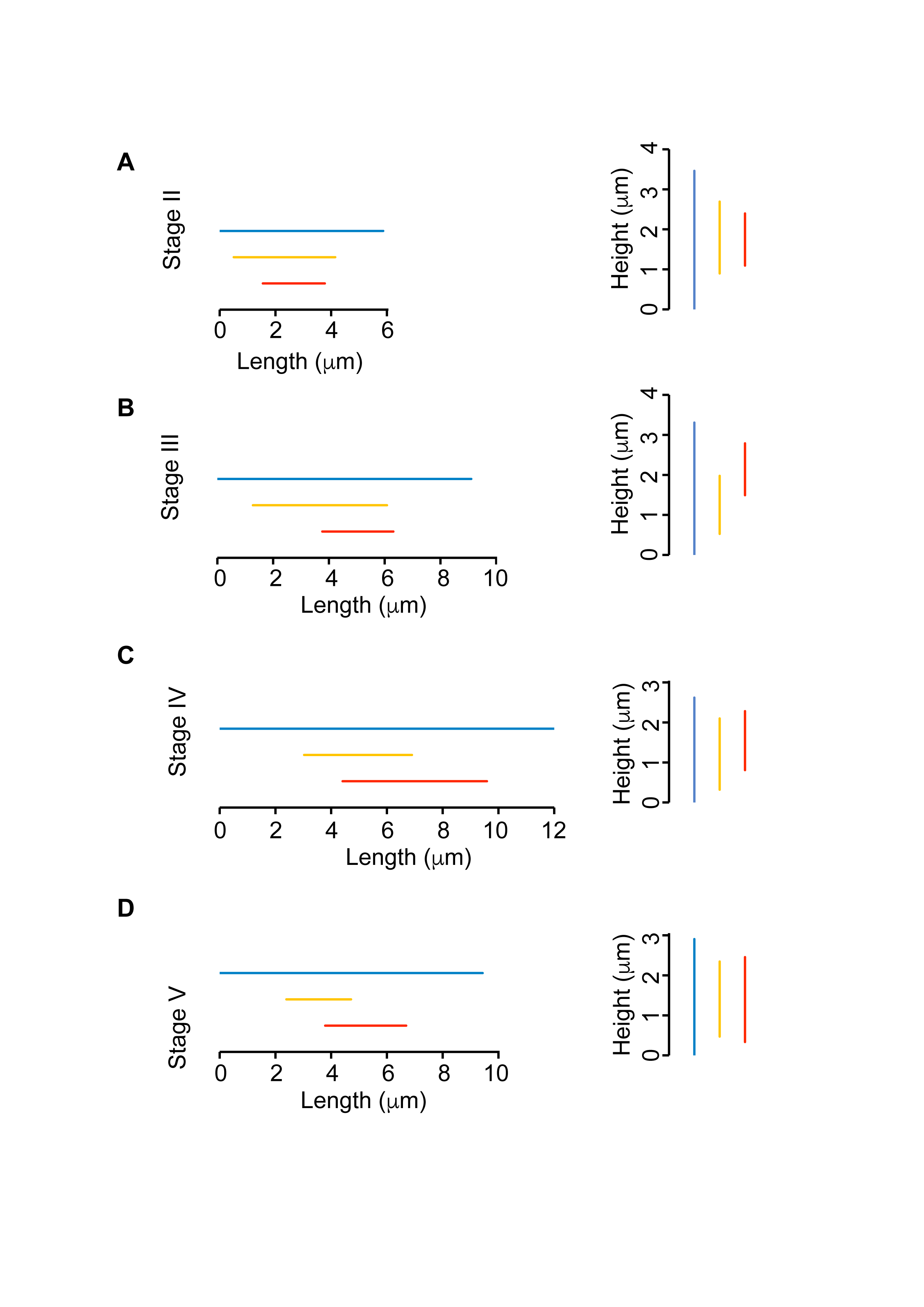

Supplement: S2 Fig — Related to Fig 1. Average lengths and heights for the parasite (blue), the nucleus (yellow) and the mitochondrion (red) at stage II (A), III (B), IV (C) and V (D). The individual measurements are provided in S1 Table. (TIF) [file ppat.1006659.s002.tif]

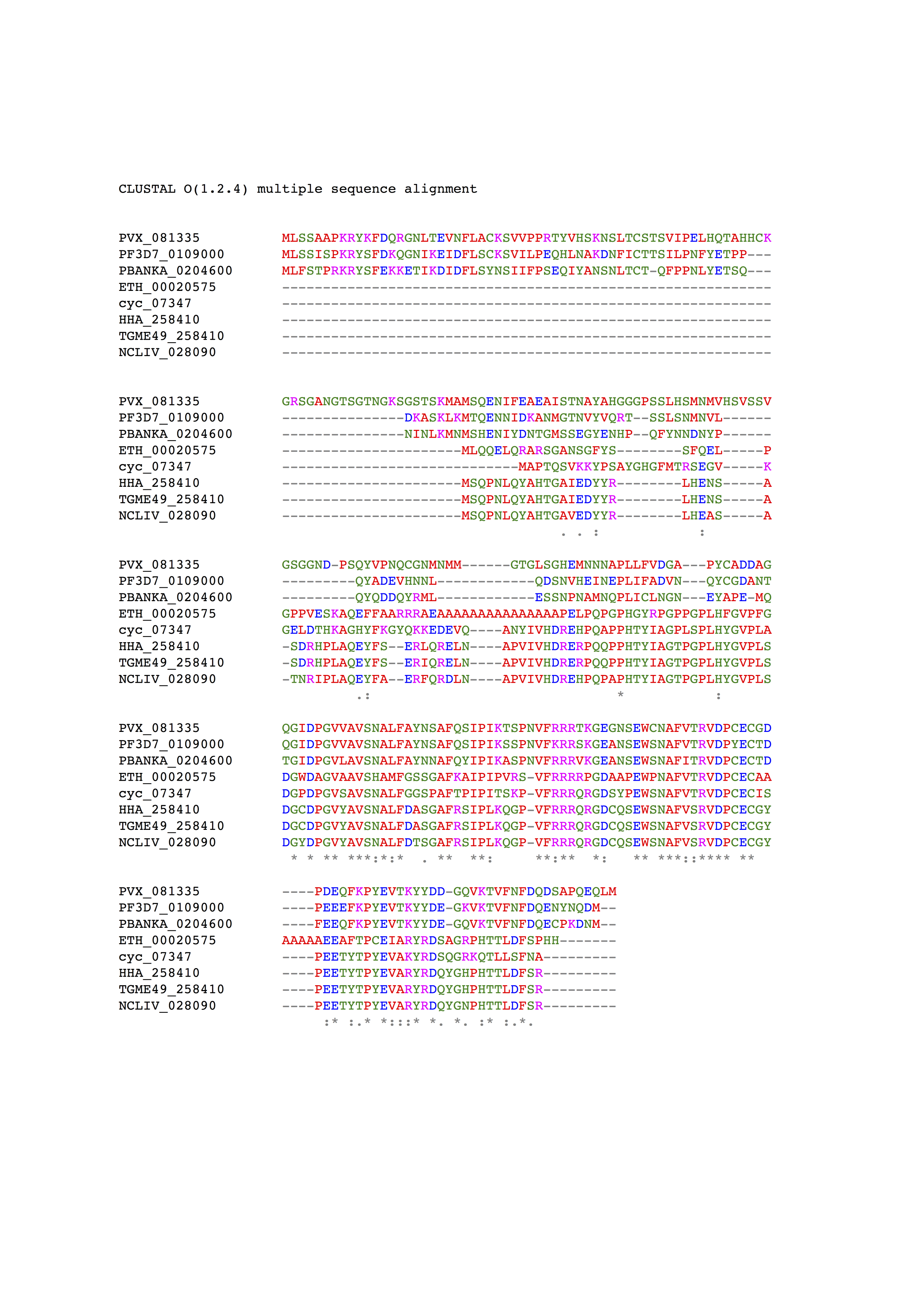

Supplement: S3 Fig — Related to Fig 3. Sequence alignment of PhIL1 homologs in Apicomplexan parasites. Sequences were obtained from the EUPath website and a multiple sequence alignment of the annotated protein sequences was performed using Clustal Omega. The following sequences were used: PF3D7_0109000, P. falciparum 3D7, photosensitized INA-labeled protein PHIL1, putative; PVX_081335, P. vivax Sal-1, hypothetical protein, conserved; PBANKA_0204600, P. berghei ANKA, photosensitized INA-labeled protein PHIL1, putative; HA_258410, Hammondia hammondi strain H.H.34, photosensitized INA-labeled protein PHIL1; TGME49_258410, T. gondii ME49, photosensitized INA-labeled protein PHIL1; cyc_07347, Cyclospora cayetanensis strain CHN_HEN01, photosensitized ina-labeled protein phil1; NCLIV_028090, Neospora caninum Liverpool, conserved hypothetical protein; ETH_00020575, Eimeria tenella strain Houghton, PhIL1, related protein. (TIF) [file ppat.1006659.s003.tif]

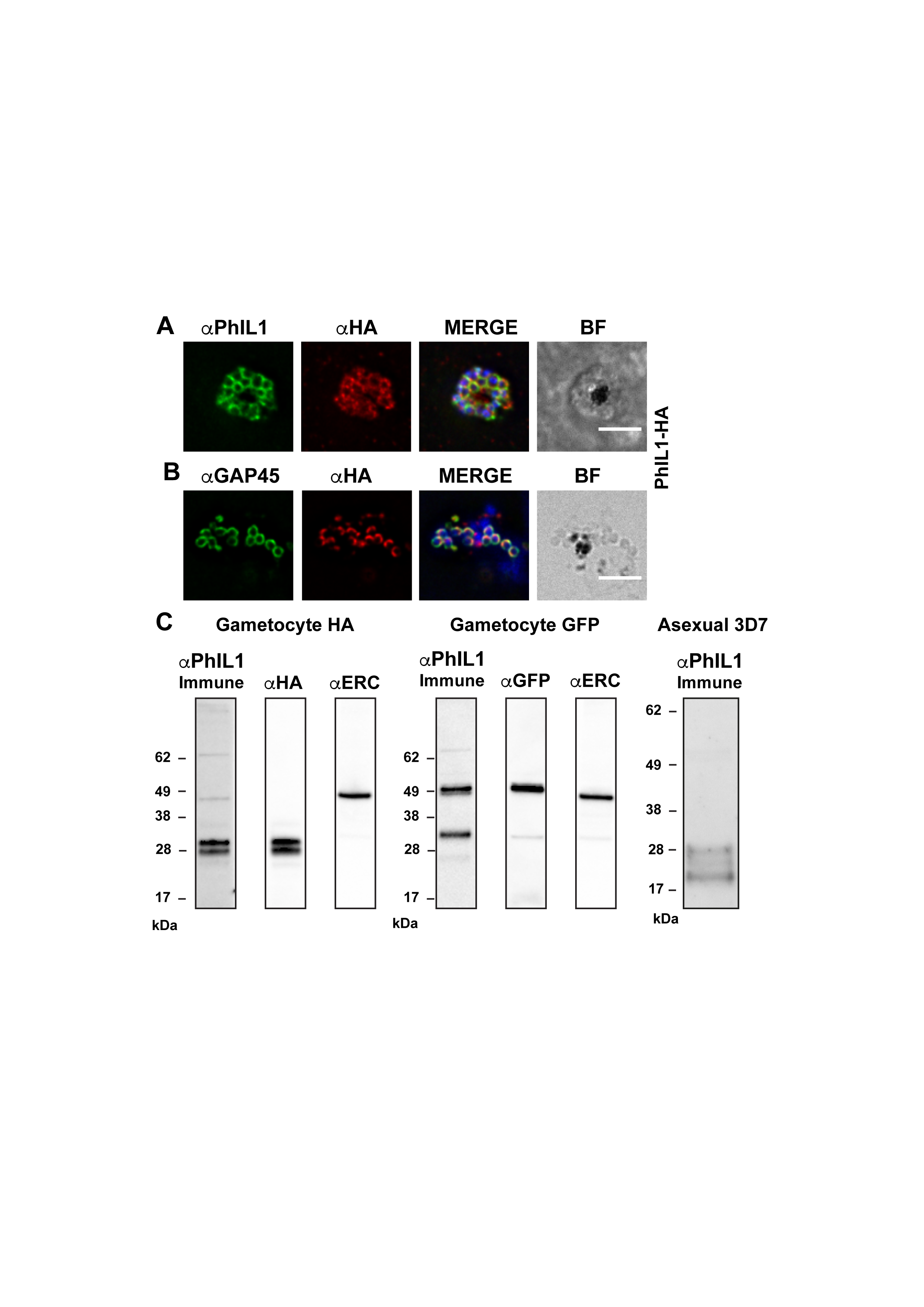

Supplement: S4 Fig — Related to Fig 3. (A,B) Immunofluorescence microscopy of schizont-stage asexual parasites (PhIL1-HA), labeled with rabbit anti-PhIL1 (green), mouse anti-HA (red) and rabbit anti-GAP45 (green). Nuclei were stained with DAPI. Scale bar: 5 μm. (C) Western blot analysis of saponin-treated pellets of stage IV gametocytes harvested from PhIL1-GFP and PhIL1-HA transfectants. The membrane was probed with anti-PhIL1, anti-HA, anti-GFP, anti-GAP45 and anti-ERC (as a loading control). Right hand side: Schizont extracts were probed with anti-PhIL1 antiserum. (TIF) [file ppat.1006659.s004.tif]

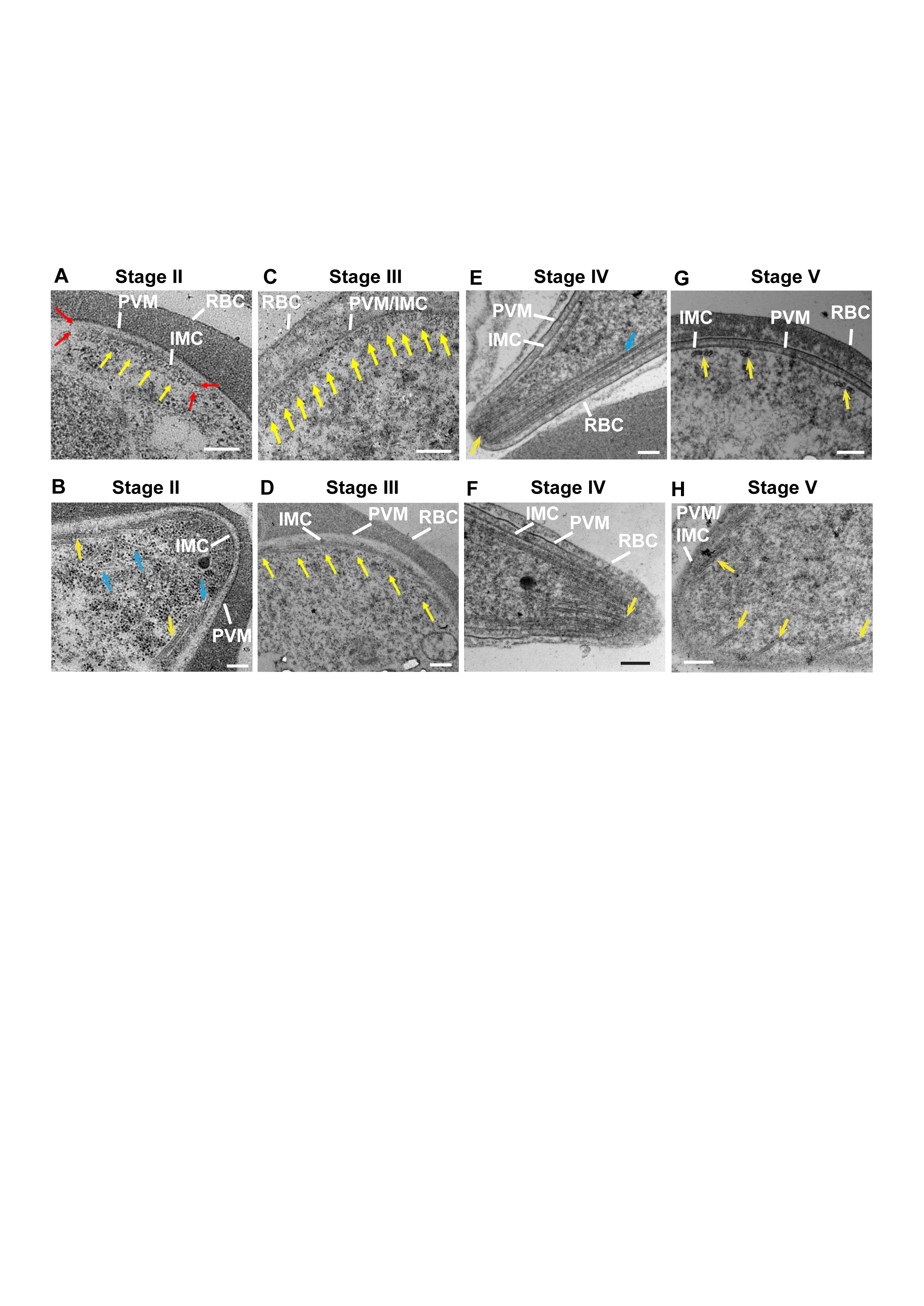

Supplement: S5 Fig — Related to Fig 4. (A) Electron micrographs of stage II gametocytes showing microtubules (yellow arrow) lying along the developing IMC (red arrows). (B) Stage II gametocyte cut longitudinally highlighting microtubules crossing the cytoplasm (blue arrows) and IMC-associated microtubules (yellow arrows). The IMC has developed ahead of the microtubule network. (C) Cross-sectional and (D) longitudinal views of a stage III gametocyte showing microtubules (yellow arrows) underneath the IMC. (E-F) Longitudinal sections highlighting the microtubules associated with the IMC along the body of the gametocyte (blue arrow) and the arrangement of the microtubule network at the tips of the gametocyte (yellow arrow). (G-H) Stage V gametocytes, showing cross-sectional (G) and longitudinal (H) views highlighting the disassembled microtubule network and the remnant stubs of microtubules at the parasite periphery (yellow arrows). The RBC membrane, PVM, and the double membrane of the IMC are indicated. Scale bars: 200 nm. (TIF) [file ppat.1006659.s005.tif]

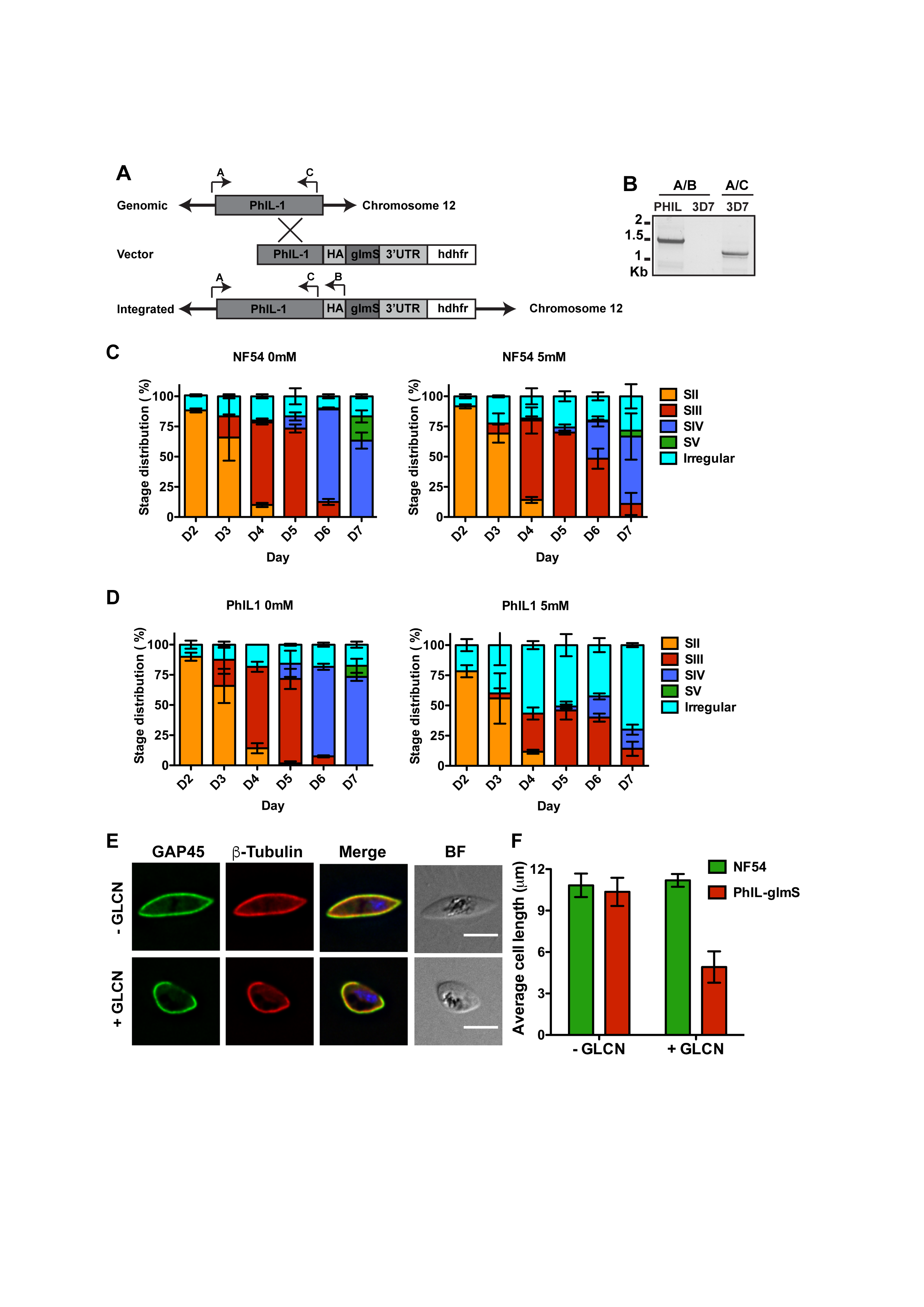

Supplement: S6 Fig — Related to Fig 5. (A) Schematic of the Phil1 genomic locus, the vector and the integrated vector showing primer placement. (B) PCR-based confirmation of integration of pPhIL1-HA-glmS into the genomic locus. Gametocyte stage progression counts from Giemsa smears taken on days 2–7 of gametocyte development for wild type NF54 (C) and PhIL1-HA-glmS (D) parasites, plus or minus 5 mM glucosamine. The percentage of each parasite stage on each day is presented. The data represent the average of 3 separate experiments. Stages are color-coded and “irregular” refers to parasites that do not fit morphologically into a gametocyte stage description. (E) Immunofluorescence microscopy confirming the presence of the IMC, labeled with anti-GAP45 (green) and microtubules, labeled with anti-β-tubulin (red), at the parasite periphery following PhIL1 knockdown. Scale bar: 5 μm. (F) Average cell length measurements for wild type NF54 and PhIL1-HA-glmS parasites plus or minus glucosamine. Data are represented as mean ± SEM; n = 11 cells. (TIF) [file ppat.1006659.s006.tif]

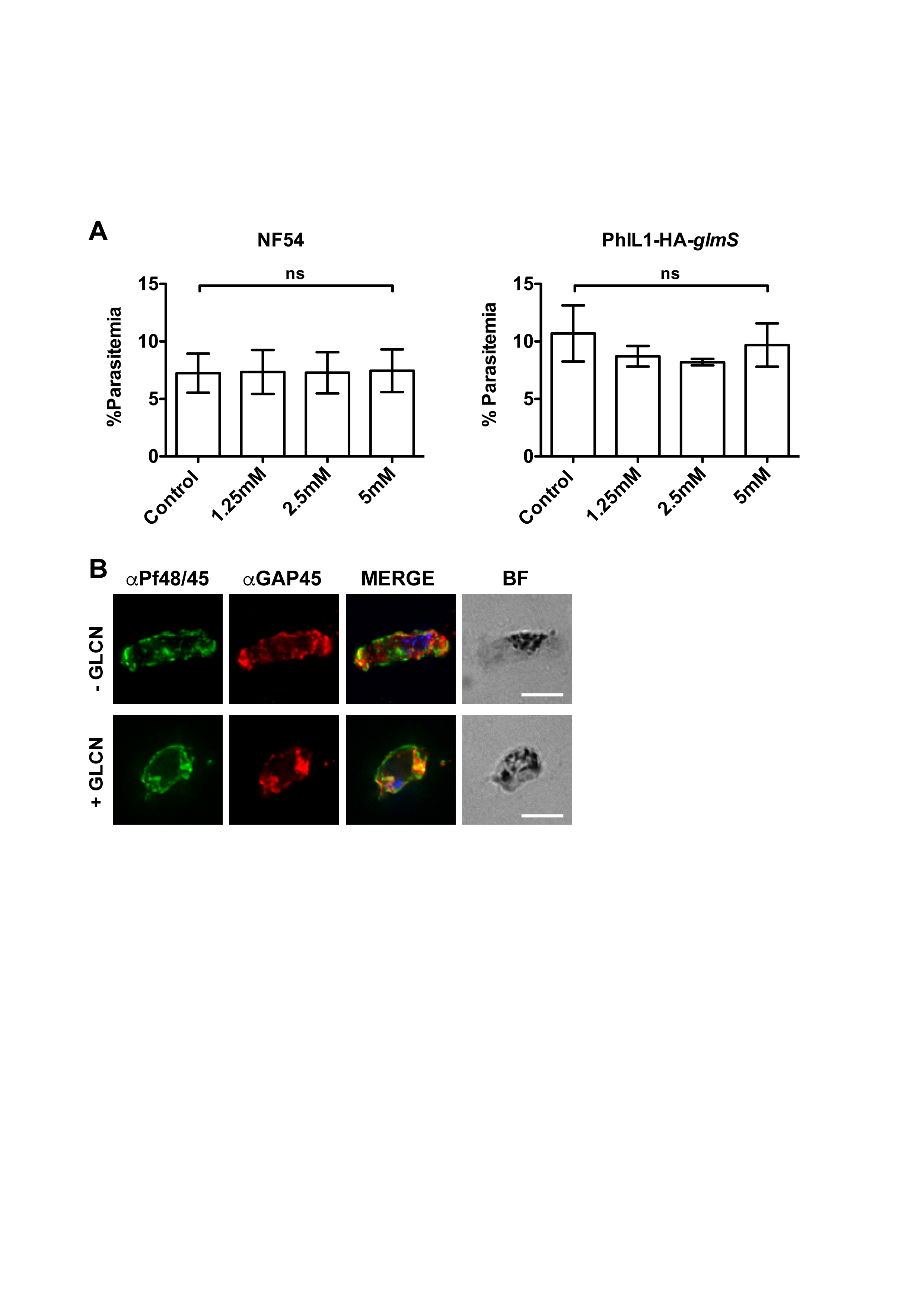

Supplement: S7 Fig — Related to Fig 5. (A) Counts of wild type NF54 and PhIL1-HA-glmS asexual parasites subjected to a range of glucosamine concentrations. Parasites were treated for 48 hours with or without glucosamine from the ring stage. The data represent the mean ± SEM for 3 separate experiments. (B) Immunofluorescence analysis of PhIL1-HA-glmS parasite at day 8 of development in treated and untreated conditions. The images reveal the presence of Pf48/45 (green) at the parasite periphery following knockdown. Staining with anti-GAP45 (red) shows that the IMC is present in both the treated and untreated samples. Nuclei are stained with DAPI (blue). Scale bar: 5 μm. (TIF) [file ppat.1006659.s007.tif]

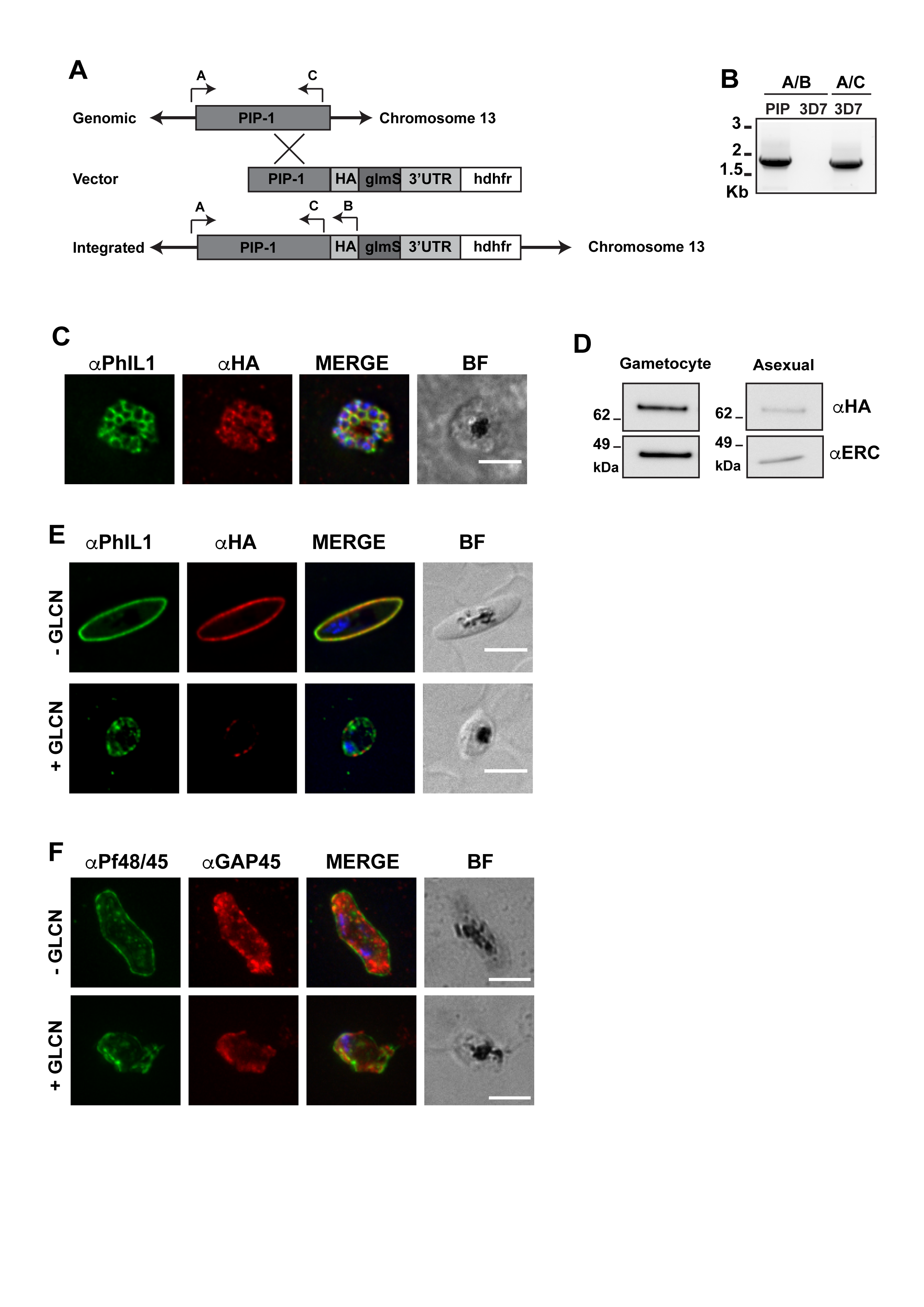

Supplement: S8 Fig — Related to Fig 7. (A) Schematic of the PIP1 genomic locus, the vector and the integrated vector showing primer placement. (B) PCR-based confirmation of integration of pPIP-HA-glmS into the genomic locus. (C) Schizonts were prepared for immunofluorescence microscopy and probed with anti-HA (red) and anti-PhIL1 (green), revealing the presence of PIP1 at the IMC. Nuclei are stained with DAPI. Scale bars: 5 μm. (D) Western analysis of PIP1-HA-glmS extracts from schizont and stage IV gametocytes, probed with anti-HA, revealing a protein of the expected size for HA tagged PIP1. ERC is probed as a loading control. (E) Immunofluorescence analysis of PIP1-HA-glmS parasite at day 6 of development in treated and untreated conditions. The images illustrate the maintenance of PhIL1 (green) at the parasite periphery following knockdown and the significant reduction of HA (red) labeling in the glucosamine-treated samples. Nuclei are stained with DAPI. Scale bar: 5 μm. (F) Immunofluorescence analysis of PIP1-HA-glmS parasite at day 8 of development, showing Pf48/45 (green) at the parasite periphery following knockdown (and in controls). Staining with anti-GAP45 (red) shows that the IMC is present at the cell periphery in both samples. Nuclei are stained with DAPI (blue). Scale bar: 5 μm. (TIF) [file ppat.1006659.s008.tif]

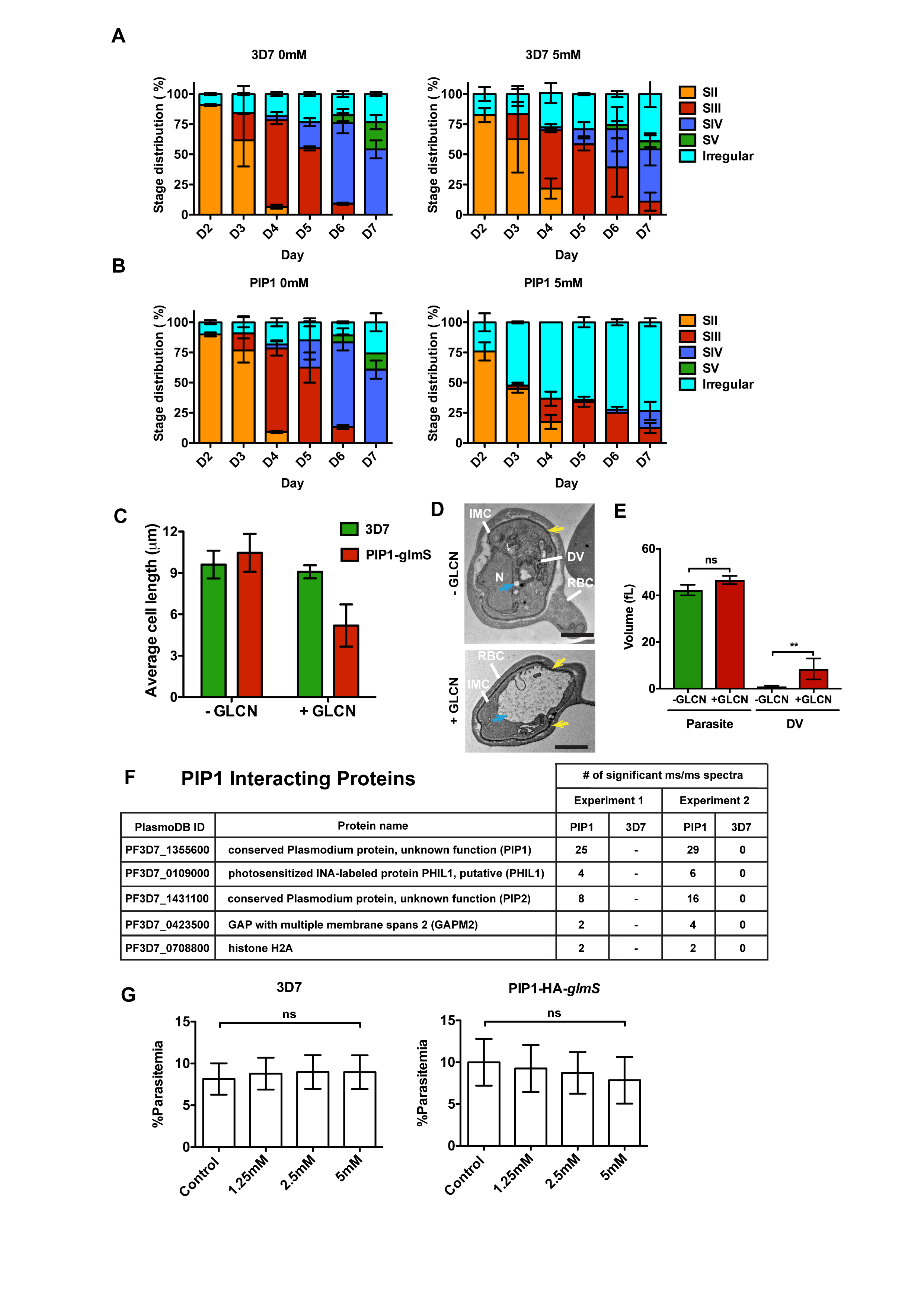

Supplement: S9 Fig — Related to Fig 7. Giemsa-stained parasite smears were used to assess development over days 2 to 7 following induction of gametocytogenesis. (A) Control 3D7 parasites were compared to (B) PIP1-HA-glmS transfectants following induction and addition of 5 mM glucosamine. (C) The length of gametocytes was assessed on day 6 after induction. Data are represented as mean ± SEM; n = 15 cells. (D) Transmission electron microscopy (TEM) thin sections (50 nm) of PIP1-HA-glmS parasites plus or minus glucosamine. The IMC is observed at the periphery of the parasite (yellow arrow). The digestive vacuole (DV) is swollen when PIP1 is knocked down (blue arrows). The IMC and RBC membranes are labeled. Scale bar: 1 μm. (E) Quantification of the SBF-SEM images. Mean volumes for the parasite and the digestive vacuole are shown. Data represent mean ± SEM. n = 5. ** P <0.01, unpaired t-test. (F) PIP1 interacting proteins. Two independent experiments were performed. Proteins that returned ≥2 significant MS/MS peptides in each experiment are included. A complete list of significant and non-significant proteins identified can be found in S2 Table. (G) Counts of wild type 3D7 and PIP1-HA-glmS asexual parasites subjected to a range of glucosamine concentrations. Parasites were treated for 48 hours with or without glucosamine from ring stage. The data represent the mean ± SEM for 3 separate experiments. (TIF) [file ppat.1006659.s009.tif]

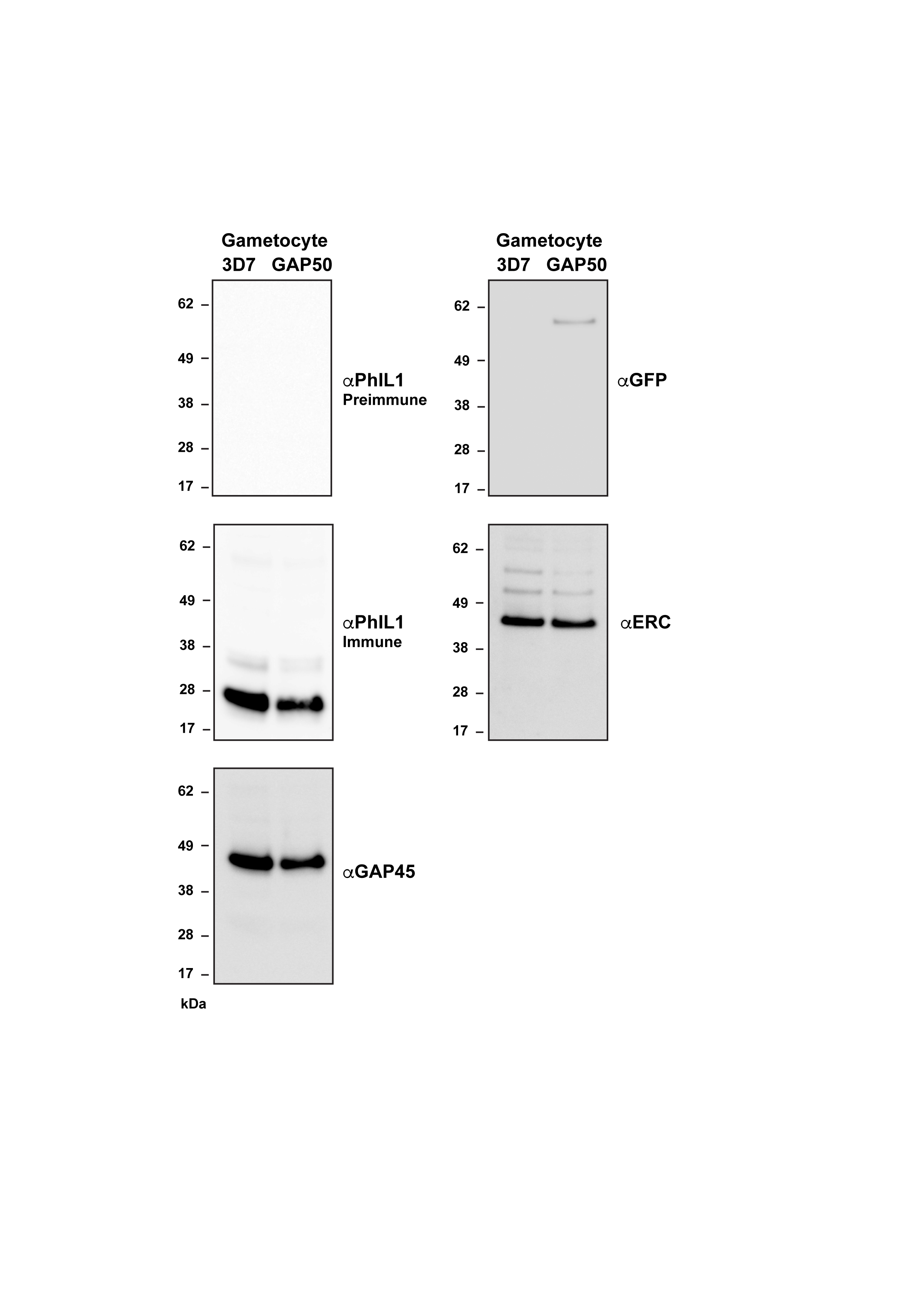

Supplement: S10 Fig — Related to Fig 3. Western blot analysis of saponin-treated pellets of 3D7 and GAP50-GFP stage IV gametocytes. Gametocytes were probed with PhIL1 pre-immune serum, anti-PhIL1 antiserum, anti-GAP45, anti-GFP and anti-ERC. (TIF) [file ppat.1006659.s010.tif]
